# The Impact of Gene Expression Variation on the Robustness and Evolvability of a Developmental Gene Regulatory Network

**DOI:** 10.1371/journal.pbio.1001696

**Published:** 2013-10-29

**Authors:** David A. Garfield, Daniel E. Runcie, Courtney C. Babbitt, Ralph Haygood, William J. Nielsen, Gregory A. Wray

**Affiliations:** 1 Department of Biology, Duke University, Durham, North Carolina, United States of America; 2 Institute for Genome Sciences & Policy, Duke University, Durham, North Carolina, United States of America; 3 Center for Systems Biology, Duke University, Durham, North Carolina, United States of America; University of Bath, United Kingdom

## Abstract

Regulatory interactions buffer development against genetic and environmental perturbations, but adaptation requires phenotypes to change. We investigated the relationship between robustness and evolvability within the gene regulatory network underlying development of the larval skeleton in the sea urchin *Strongylocentrotus purpuratus*. We find extensive variation in gene expression in this network throughout development in a natural population, some of which has a heritable genetic basis. Switch-like regulatory interactions predominate during early development, buffer expression variation, and may promote the accumulation of cryptic genetic variation affecting early stages. Regulatory interactions during later development are typically more sensitive (linear), allowing variation in expression to affect downstream target genes. Variation in skeletal morphology is associated primarily with expression variation of a few, primarily structural, genes at terminal positions within the network. These results indicate that the position and properties of gene interactions within a network can have important evolutionary consequences independent of their immediate regulatory role.

## Abstract 2

Changes in the nature of gene interactions during development help explain the robustness of early development and the basis for developmental evolution.

## Author Summary

Animal development is highly stereotypic in the face of changing environmental conditions and individual genetic differences. At the same time, developmental processes can evolve rapidly, suggesting that selectable genetic variation is hidden beneath this apparent stability. To better understand the relationship between these seemingly opposed properties of robustness and evolvability, we measured how natural variation in gene expression propagates across a network of interacting genes underlying early development in sea urchins. We found that gene interactions are not equal across development: expression variation is well buffered during early development by the use of on/off switch-like (rather than continuously tunable) regulatory mechanisms, while during later development it has a greater impact on the expression of downstream target genes and on morphology. Using a breeding design, we were able to detect a substantial genetic component to the observed variation in gene expression. Interestingly, the degree of genetic contribution was greatest during early development and specifically at points of switch-like regulation, suggesting that the properties of developmental gene regulatory networks that underlie robustness also promote the accumulation of genetic variation that could seed adaptation.

## Introduction

The process of development is a balancing act in an unpredictable world: it is often remarkably resilient, producing consistent phenotypes in the face of new mutations and environmental perturbations, but it is also adaptable, allowing for the evolution of novel phenotypic traits in response to environmental change. The first property is essential for the survival of individuals and the second for the persistence of populations. Yet these two critical aspects of development seem diametrically opposed, since buffering stabilizes phenotypes while adaptation permanently changes them [1]–[3]. An outstanding challenge for both systems biology and evolutionary biology is understanding the molecular mechanisms that allow development to buffer phenotypes while retaining flexibility [4],[5].

Key to understanding both buffering and adaptation is measuring how, and to what extent, variation in developmental gene function impacts downstream phenotypes [6],[7]. At its core, buffering must result from reduced variation in underlying developmental processes such as cell fate specification and morphogenesis. Although the detailed molecular mechanisms underlying developmental buffering are not clear, they likely involve redundancy, modularity, feedback loops, and threshold (nonlinear) interactions among regulatory molecules that reduce the downstream consequences of variation within regulatory networks [8],[9]. The extent to which variation in molecular processes such as transcription, splicing, translation, and phosphorylation during early development affects later processes and, eventually, organismal phenotypes remains poorly understood. Measuring such variation during development is also critical for understanding adaptation, as it provides the raw material for natural selection. Several well studied cases demonstrate that alleles segregating in wild populations influence morphology in ecologically significant ways by changing specific regulatory interactions during development that in turn alter gene expression [10]–[12]. It remains unclear, however, whether such alleles are common within natural populations and how they are able to influence morphology despite buffering during development.

In this study, we examine three pertinent questions for understanding the relationship between developmental buffering and evolvability: How much variation in the expression of developmental regulatory genes exists within a natural population? What impact does this variation in gene expression have on downstream genes within a regulatory network? And finally, how does expression variation during development influence the morphological phenotypes that lie at the interface between organism and environment and are therefore potential targets of natural selection?

**Figure 1:**
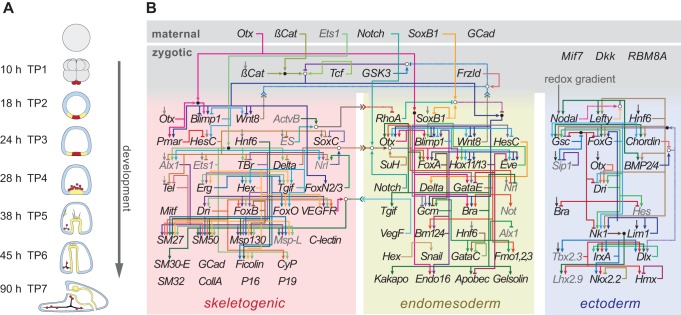
Developmental gene regulatory network of *S. purpuratus*. (A) Development progresses from the egg (top), through cleavage and gastrulation (middle), to a free living larva capable of feeding (bottom). Skeletogenic cell lineage indicated in red, skeleton in solid black. The seven post-fertilization stages and times (hours) shown correspond to time points 1–7 discussed throughout this article. (B) The gene regulatory network is initiated by maternal transcripts and proteins (top) that activate a cascade of subsequent gene regulatory interactions (see text for references). Names of genes assayed in this study are shown in black, other genes in gray. Solid lines, colored to allow visual separation, denote experimentally verified direct molecular interactions among genes: transcription factor:DNA binding (arrows = activators, bars = repressors) or ligand:receptor interactions (nested arrowheads pointing to receptor). Distinct spatial territories of cell fates specific in the embryo are indicated by colored background and name. Based on [13]–[24].

To address these questions, we measured naturally occurring variation in gene expression within a well-defined developmental gene regulatory network, as well as the impact of expression variation on downstream gene expression and on morphology. The network of >100 genes that we examined (Figure 1) contains all of the genes known to be involved in axial and cell type specification during embryogenesis in the purple sea urchin, *S. purpuratus* [13]–[24] as well as many of the important genes involved in mesoderm specification and the formation of the larval skeleton. Most of the >200 connections in this network are direct intermolecular interactions identified from extensive experimental studies including knock-downs, over-expression assays, cell transplantations, protein:DNA binding assays, and transient transfection assays. The network thus provides a detailed account of gene regulatory interactions necessary, though not sufficient, for early development.

Three additional features make this gene regulatory network particularly useful for studying the consequences of variation in gene expression during development. First, the network spans the major phases of development: from the unfertilized egg, through embryonic patterning and cell fate specification, to morphogenesis and terminal cellular differentiation. It thus offers an opportunity to examine how changes in the expression of regulatory genes influence phenotypes throughout development. Second, the network includes all of the regulatory genes and many of the structural genes known to be involved in the formation of the larval skeleton, a discrete and readily imaged three-dimensional structure [25]. This allows one to quantify covariation between molecular processes and the morphological structures they produce within the context of a known topology of largely direct regulatory interactions (Figure 1). Because variation in the size and shape of the larval skeleton influences fitness [26]–[30], it provides a relevant phenotypic readout of the consequences of variation in gene expression throughout the network. Third, wild populations of *S. purpuratus* harbor high levels of genetic diversity [31],[32], including substantial variation within cis-regulatory elements that mediate regulatory interactions within the network [33]–[36]. This genetic variation provides a natural source of perturbation that allows one to measure the impact of functional variation on developmental processes and morphology. Because these perturbations derive from natural rather than induced mutations, they are directly relevant to understanding the operation and evolution of development in the wild.

We measured variation in gene expression throughout the gene regulatory network at multiple developmental time points in crosses of outbred individuals of *S. purpuratus* collected from a natural population. We also measured the phenotypic impact of this variation, both on downstream gene expression and on the resulting morphology of the larval skeleton. While several previous studies have examined correlations between natural variation in gene expression and ecologically significant traits from single genes [10],[12],[37],[38] or within short pathways [6],[39],[40], this is the first study we are aware of that has sought to quantify expression variation throughout an extensive gene regulatory network spanning development from embryogenesis to the production of organismal traits, and to relate variation in gene expression throughout a developmental network and across developmental stages to specific morphological trait consequences.

## Results

In order to examine the extent and consequences of variation in gene expression within the gene regulatory network, we set up a 6×6 cross using outbred parents derived from the same wild population. We raised the 36 families as replicated cultures in a randomized design within a growth chamber at the Duke University Phytotron and sampled individuals from each culture at seven time points during development (Figure 1A). From these samples we measured transcript abundance in pooled samples of several hundred embryos for 74 interacting genes within the network using DASL, a multiplexed amplification assay, on the Illumina BeadStation (see Methods). At time point 7 we also quantified morphological variation in the larval skeleton using standard landmarks in ∼20–30 individuals from each family. These data form the basis of the analyses detailed below.

### Variation in Developmental Gene Expression Has a Significant Genetic Component

Variation in developmental gene expression within a population can arise from many sources, including genetic differences and non-genetic parental influences such as egg quality, which contribute to resemblance among relatives, as well as from environmental influences and stochastic processes, which do not. Because the cultures we analyzed were derived from a controlled cross with known parents (the NCII breeding design), we were able to estimate the magnitude of genetic and non-genetic parental contributions (collectively, parental effects), based on correlations in expression levels among related individuals relative to the population as a whole. In the NCII design, male and female contributions each provide a direct estimate of the additive (heritable) genetic contribution to gene expression variation [41]. In the case of the male effects, the estimate is direct, as sperm contribute to offspring phenotypes almost exclusively through genetic effects. In the case of maternal effects, however, estimates can be distorted by differences in additional maternal contributions such as mRNA or nutrients loaded into the egg (which may themselves have a genetic component). For a variety of reasons (discussed below and in Text S1), we believe non-genetic maternal effects to be relatively minor subsequent to the first time point we examined.

We observed pervasive parent-of-origin effects on gene expression throughout development. The expression levels of most genes in the network (72/74) showed significant paternal and/or maternal effects at one or more of the time points sampled. For most of these genes, we could ascribe a direct contribution from genetic variation at multiple time points (Table S1): some 70% of the genes (52/74) showed significant paternal effects, evidence of widespread genetic influences on quantitative variation in gene expression, since paternal effects are likely to be largely genetic [41].

**Figure 2:**
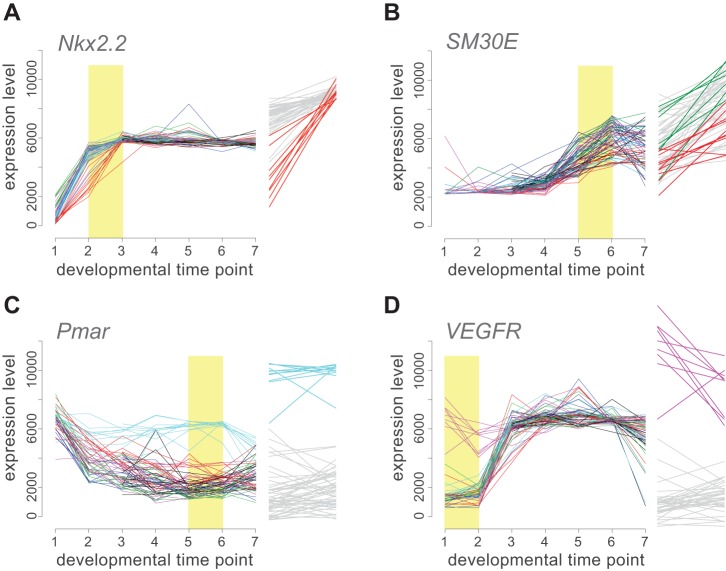
Parent-of-origin effects on gene expression profiles. Changes in transcript abundance across seven developmental stages are plotted for each family for four representative genes. Families are color-coded by parent of origin: dam in (A, C, D) and sire in (B). In each case, gene expression profiles in the families derived from one parent stand out as distinct from all the other families (color versus grey in magnified time segments to the right of each plot; yellow rectangles indicate magnified portion).

In most cases, the magnitude of variation explained by parent-of-origin was modest, consisting of quantitative differences in the timing or level of gene expression on the order of 10%–15% relative to mean expression level among families (Table S1). For instance, up-regulation of *Nkx2.2*, which encodes a transcription factor, was delayed in offspring of one female relative to other families despite reaching typical levels later (Figure 2A), while expression levels of *SM30-E*, which encodes a dominant component of the biomineral matrix of the skeleton, were on average higher and lower in offspring of two different males relative to other families (Figure 2B).

In a few cases, differences in gene expression among families were more substantial. The key regulatory gene *Pmar*, whose product forms part of a double-negative gate that restricts expression of skeletogenic genes to a subset of cells in the early embryo [42], was expressed at sustained high levels in the offspring of one female long after it was barely detectable in other families (Figure 2C). Transcripts of *VEGFR*, which encodes a receptor that mediates critical cell fate decisions [43], were abundant in the very early embryo in offspring of just one female (Figure 2D), perhaps reflecting maternal loading of transcripts. Because we assayed gene expression in whole embryos, we cannot distinguish whether these differences involved increased expression within their normal spatial domains or ectopic expression involving additional cells. What is clear, however, is that there was no detectable impact of these two anomalous expression profiles on the subsequent expression of known downstream targets, even though *Pmar* and *VEGFR* both encode key regulators of the skeletogenic portion of this regulatory network.

### Genetic Contributions to Expression Variation Change during Development

**Figure 3:**
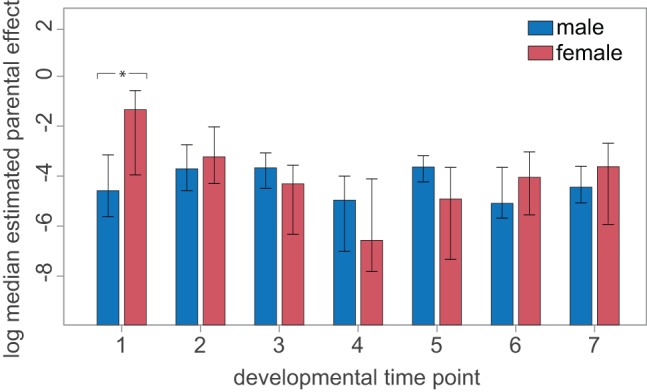
Parental components to gene expression variation. Median female and male parental contributions to scaled expression variation (variance/mean^2^; see Methods) are plotted for each time point on a log scale to facilitate visualization (analyses were carried out on untransformed values using non-parametric tests); whiskers on each bar indicate quartiles. Maternal effects significantly exceed paternal effects only at the first time point and paternal effects are relatively uniform across development.

Zygotic transcription occurs at very low levels during the first few cell cycles after fertilization in *S. purpuratus* and increases dramatically by 4th–5th cleavage [44],[45]. This corresponds approximately to our time point 1, which should thus show strong maternal contributions to variation in transcription levels. Beginning with time point 2, maternal and paternal genetic contributions should be equal in magnitude, and any significantly larger maternal effects would constitute evidence for maternal-specific genetic and non-genetic (e.g., environmental) contributions to gene expression variation [41]. Consistent with an important role for genetic influences on gene expression, we found that maternal effects were significantly greater than paternal effects only at the first time point (*p* = 0.004, Wilcoxon) (Figure 3). In contrast, the distribution of paternal effects remained fairly constant across developmental time points, with an important contribution to the average parental effect at each time point coming from a few transcripts with relatively high paternal contributions (Figure S1B). Furthermore, we observed a shift from a modest, though significant, correlation between the magnitude of maternal and paternal effects at time point 1 (Spearman Rho = 0.54, *p* = 4×10^−5^) to higher correlations at later time points (Spearman Rho = 0.87, *p* = 2.2×10^−5^), consistent with predominately zygotic transcription after the first time point.

The magnitude of parental influences on variation in gene expression clearly changed during development (Kruskal-Wallis, *p* = 2.9×10^−5^, χ^2^ = 61.21, df = 6) (Figures 3 and S1B), with the first two time points showing significantly greater variation in parental effects among families than later time points (mean σ^2^_est_ = 0.286 versus 0.064; Wilcoxon, *p* = 3.48×10^−8^, W = 32,208) (Figure S1A). Differences in the magnitude of parental variances among time points remained significant even after excluding time point 1 (Kruskal-Wallis, *p* = 2.4×10^−3^, χ^2^ = 18.44, df = 5), suggesting that the zygotic component of genetic influences on variation in gene expression changes throughout development and that genetic contributions to gene expression are at least as great during early development as they are during morphogenesis. Importantly, we obtain qualitatively similar results when we examine only statistically significant paternal effects (Kruskal-Wallis, *p* = 1.5×10^−3^, σ^2^_est_ = 0.038 versus 0.026 *p* = 0.09 Wilcoxon) or when we look at mean parental contributions for genes only at the times in which they are known from prior research (see above) to be involved in direct regulatory interactions (protein:DNA and protein:protein) (Kruskal-Wallis, *p* = 0.039, σ^2^_est_ = 0.18 versus 0.07 *p* = 0.051 Wilcoxon).

Taken together, these results suggest that this wild population harbors substantial amounts of genetic variation that influence gene expression during even the earliest stages of embryonic and larval development. This finding is consistent with the high levels of genetic variation in known cis-regulatory sequences that previous studies have documented in wild populations of *S. purpuratus*, including SNPs within experimentally validated transcription factor binding sites regulating the expression of *FoxB*, *Endo16*, and *SM50* [33]–[36].

### The Nature of Regulatory Interactions Changes during Development

In order to understand the impact that variation in the expression of regulatory genes has on downstream targets, we first examined correlation coefficients (r^2^) between pairs of genes for which there is experimental evidence of a direct regulatory interaction. We restricted these analyses to time points when each interaction is known to occur (http://sugp.caltech.edu/endomes/; no data are available for time point 7). The information in this database has been painstakingly compiled, and represents arguably the most complete picture of a developmental gene regulatory network to date [13]–[24]. It is not, however, perfect: most noted regulatory interactions are likely to be real, but many others remain to be identified. This has a minor impact on the comparison of correlation coefficients between known interactors as compared with random pairs of genes thought not to interact. However, inadvertently including some actual interactions in the background model makes the test more conservative, so this is not a major concern.

**Figure 4:**
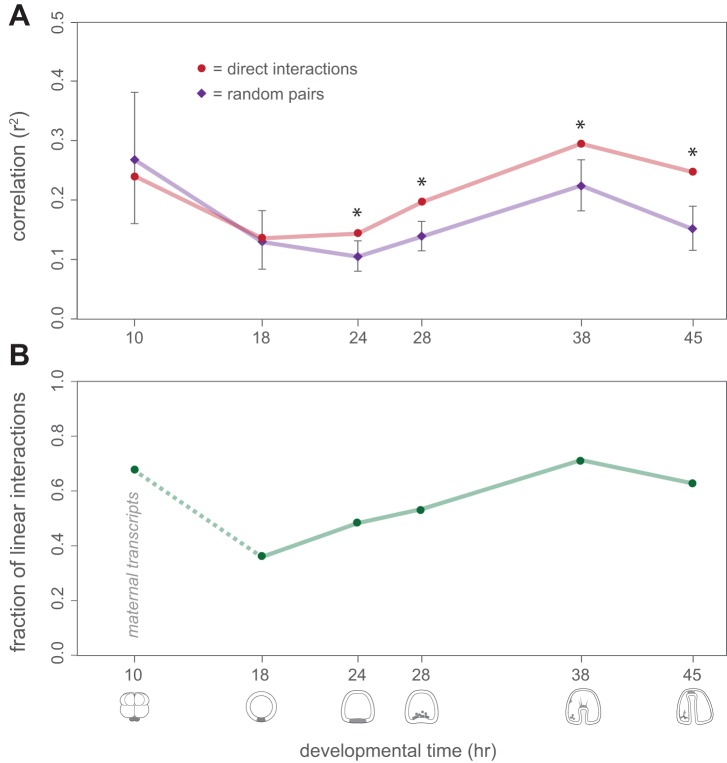
Changes in regulatory interactions across development. (A) Mean correlation (r^2^) in expression between pairs of genes are plotted for known direct regulator-target interactions (red) and random pairs of genes not thought to interact (purple). Error bars for random interactions are based on boot strap replicates carried out independently for each stage; values for known interactors are counts at each stage and thus no error bars are shown. Asterisks denote significant differences between interactors and random pairs of genes (*p*<0.01, by permutation, for all but time point 3, for which *p*<0.05). Note that known interactors are no more correlated than random pairs of genes during early development, but become more highly correlated from time point 3 onwards. (B) The proportion of sensitive regulatory interactions among known interactors is plotted. Many regulatory interactions among zygotically expressed genes are insensitive (i.e., switch-like) during embryogenesis, with an increasing proportion of sensitive (i.e., quantitative) interactions at later time points.

As expected, correlations over all stages were, on average, significantly stronger between genes that are known to interact than between genes with no known regulatory interactions (*p*<0.01, permutation). However, the strength of these correlations changed dramatically during development. During the first two time points, r^2^ values were no greater, on average, for interacting genes (whether via protein:DNA or protein:protein interactions) than for non-interacting pairs (Figure 4A). In contrast, correlations rose significantly during the subsequent time points, suggesting that functional dependencies between genes become quantitatively stronger and more tightly correlated later in development (*p*<0.01 for each of time points 3–6, permutation). Importantly, correlations among pairs of genes expressed in the same tissue were often negative and were not, on average, greater than those between random pairs of genes (Figures S2 and S3). Changes in correlations over development are thus unlikely to stem from differences in tissue composition or developmental rates among broods.

**Figure 5:**
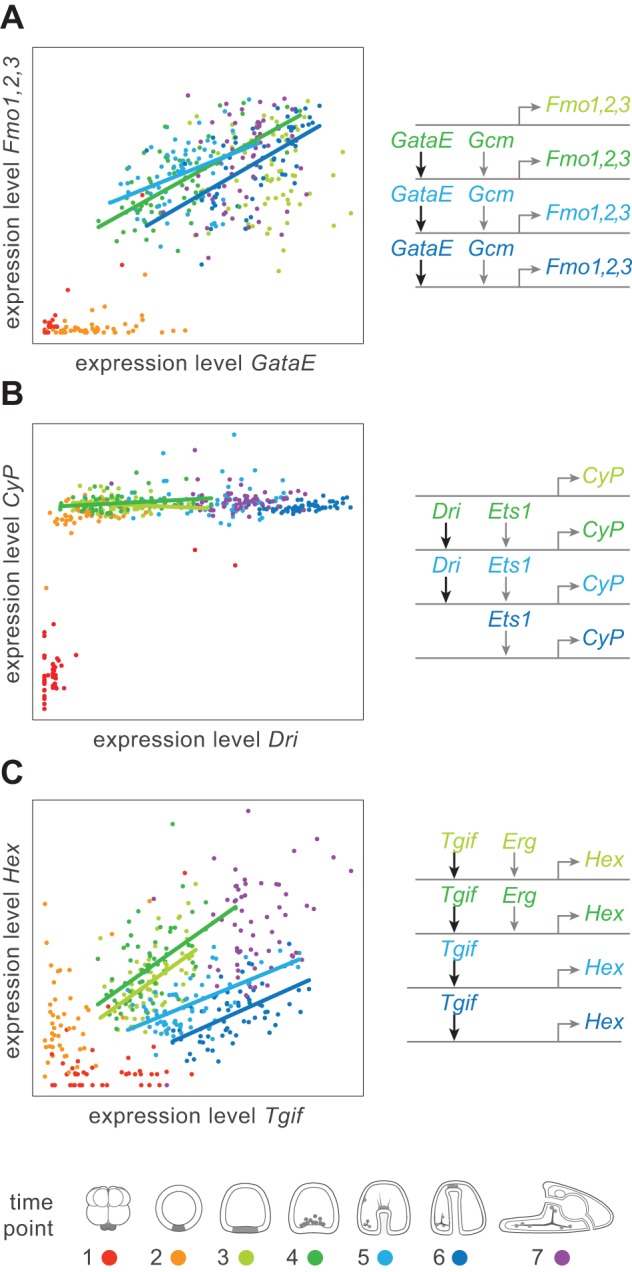
Correlations in gene expression levels among direct interactors. Scatter plots of expression levels for pairs of upstream regulators (*x*-axis) and direct targets (*y*-axis). Expression levels and regulatory interactions are color-coded by developmental time point (bottom). Regression lines are shown at active time points. Regulatory inputs to the downstream gene at active time points are drawn to the right of each plot using the same color-coding for time points. Information about active/inactive edges is not available for time point 7, which is therefore omitted. Note that gene regulatory interactions differ qualitatively. (A) Some are roughly linear: increased expression of the upstream gene (GataE) is correlated with increased expression of its direct target (Fmo1,2,3). (B) Other interactions are more switch-like: beyond a certain level, differences in expression of the upstream gene (Dri) has little impact on the expression level of its direct target (CyP). (C) Regulatory interactions can also change during development. Expression of Hex is sensitive to Tgif expression levels during all four active time points, but is more sensitive (steeper slope) when also receiving input from Erg (time points 3 and 4) than when it is not (time points 5 and 6).

Regulatory interactions also differed qualitatively during development. Some gene pairs showed strong dependencies throughout development (e.g., GataE→Fmo1,2,3, Figure 5A), suggesting that the expression level of the downstream target was sensitive to variation in the expression level of its immediate upstream regulator. In other cases, the expression of downstream genes appeared largely insensitive to quantitative variation in a direct upstream regulator beyond some threshold level of expression (e.g., Dri→CyP, Figure 5B). This second example suggests a situation in which the downstream gene is effectively buffered from variation in the expression level of the upstream regulator, at least within the bounds of normal variation encountered in the wild population.

On the basis of these patterns of correlation, we next classified each experimentally validated pair-wise regulatory interaction as being either sensitive (expression levels of the two genes show a statistically significant correlation; e.g., Figure 5A) or insensitive (switch-like or Boolean; no quantitative relationship between expression levels above a threshold; e.g., Figure 5B). Because the quantitative and qualitative nature of a regulatory interaction can change during development (Figure 5C), we analyzed each interaction independently at each time point at which it occurs. To ensure an equal power to detect sensitive edges at each stage of development, we quantified overall levels of variation at each time point (Table S2) and observed no relationship between these numbers and the number of edges classified as sensitive/insensitive. Furthermore, because the average expression levels of the genes used in this test were approximately equal at each time point, we can rule out influences from changes in the accuracy of expression measurements between time points.

Interestingly, the relative proportion of sensitive and insensitive edges changed over time (Figure 4B; χ^2^ = 10.91, *p* = 0.032). The proportion of sensitive edges increased substantially from early to later stages of development when considering zygotic transcription only. The first time point marks an exception. However, at this time a large proportion of transcripts present are still maternally derived [44],[45], and covariances between genes are both significantly greater and more structured than at subsequent stages of development as revealed by comparisons of genetic covariation matrices across time points (see Methods and Text S1). Correlations among genes at this earliest time point we sampled are thus likely confounded by differences in maternal provisioning among females affecting sets of genes in unison rather than causal relationships among interacting genes in the network, a result we discuss further below.

Insensitive regulatory interactions may allow more genetic variation to accumulate within the population by buffering downstream phenotypes from the consequences of mutations impacting the expression of upstream genes. To test this possibility, we compared the size of paternal effects, the most conservative estimator of genetic influences [41], for genes upstream of insensitive versus sensitive regulatory interactions. We observed significantly greater paternal effects upstream of insensitive interactions than sensitive ones (mean σ^2^_paternal_ = 0.029 for insensitive versus 0.016 for sensitive, *p* = 0.037, Kolmogorov-Smirnov). In order to account for changes over developmental time in average paternal effects and in the proportion of insensitive interactions, we repeated the analysis incorporating the effects of developmental time point in the model and the result was still significant (*p* = 0.048). Thus, insensitive regulatory interactions may contribute to buffering and may influence the distribution of genetic variation across the network.

### Variation in Gene Expression Influences Skeletal Morphology

**Figure 6:**
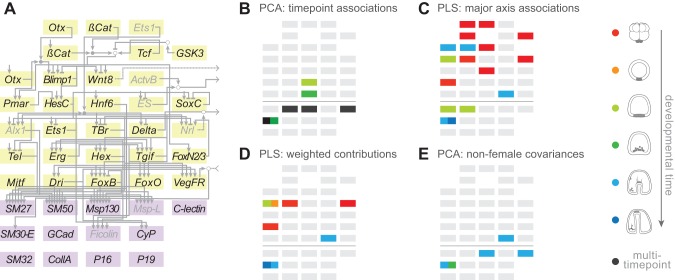
Correlations between gene expression and larval morphology. (A) Gene regulatory sub-network in skeletogenic cells (see Figure 1 for broader network context). Yellow boxes indicate genes encoding regulatory proteins; purple boxes indicate genes encoding structural proteins of the skeleton and surrounding matrix. These boxes correspond to rectangles in the remaining panels, with a horizontal line separating the two classes of genes. (B–E) Results of tests for correlations between variation in gene expression and variation in skeletal morphology. Gray indicates no correlation; color indicates correlation with expression from a single time point; black indicates a correlation based on multiple time points (see Text S1). (B) Morphological associations with expression based on PCA. SM30-E is related to PC I (primarily length), FoxB and Hex with PC III (primarily aspect ratio). (C) Morphological associations based on partial least squares analysis. Very early effects (time point 1) operate through regulators high in the network. (D) Morphological associations based on weighted contributions by partial least squares analysis. Four genes show associations from early stages and three from late stages. (E) Morphological associations that are conservatively based solely on male genetic contributions. The three strongest associations come from late expression. Note that SM30-E is identified in all four analyses.

While gene expression is an important intermediate phenotype, the ultimate products of developmental gene regulatory networks are the morphological and physiological traits upon which natural selection directly acts. To better understand how natural variation in gene expression within the network influences ecologically relevant traits, we next examined associations between variation in gene expression throughout the network and variation in the morphology of the larval skeleton. Because the size and shape of the larval skeleton is closely associated with feeding rates and survivorship [27]–[29], morphological variation in this structure (Figure 6A) is likely to be subject to natural selection.

After measuring the skeletons of larvae from each culture at time point 7 using established morphological landmarks [46],[47], we carried out a principle components analysis (PCA), and found that the first three principle components collectively explained ∼89% of overall variation in size and shape (Table S3). These factors showed strong evidence of parental effects, particularly maternal effects (Table S4), as one might expect given the evidence for a relationship between variation in maternal egg quality and skeletal shape in echinoderms [47],[48]. For each gene at each time point, we then calculated Pearson's correlation coefficient between transcript abundance and each of these first three principal components. After correcting for multiple comparisons, eight genes showed statistically significant associations between gene expression at one or more time points and one or more of the principle components of skeletal variation (Figure 6B; Table S5; Text S1). Although our gene expression measurements were based on whole embryos, five of the eight genes are transcribed only in skeletogenic cells at the time points we examined, suggesting that their effects on skeletal variation are exerted through this cell type alone.

Six of the genes reside within the skeletogenic subnetwork: three (*SM30-E*, *Msp130*, *SM50*) encode abundant protein components of the biomineral matrix of the skeleton [25],[49], another (*C-lectin*) encodes one of the most abundant proteins in the cells that secrete the skeletal matrix [50], and two (*FoxB* and Hex) encode transcription factors that are direct activators of the four structural genes just mentioned [14]. The remaining two genes, *Dkk* and *Su(H)*, are regulators of Wnt and Notch signaling, respectively [20],[51], whose functions in sea urchin development have primarily been studied before the onset of skeletogenesis. Interestingly, Dkk is an abundant component of the phosphoproteome of adult skeletal matrix [49], raising the possibility that it has a more direct role in skeletogenesis.

Because the majority of associated genes (five of eight) are expressed exclusively within skeletogenic cells, correlations between their expression and skeletal morphology could be explained by differences in the number of skeletogenic cells among families. For two reasons, this is unlikely to be the case. First, as mentioned above, correlations in the expression of skeletogenic cell genes among families are no greater than background, and are often negative. Second transplant experiments that artificially increase skeletogenic cell number by more than 2-fold, which is far outside the normal range of variation, have no measurable impact on the size or shape of the larval skeleton [52],[53], arguing against a direct link between skeletogenic cell number and skeletal morphology.

These results indicate that: (1) natural variation in the expression level of several genes within the network has an impact on an ecologically important structure in the larva; (2) the genes with the largest impact are located at the termini of this gene regulatory network; and (3) these genes primarily encode cell type-specific structural proteins and their immediate regulators.

### The Impact of Variation in Gene Expression on Skeletal Morphology Changes during Development

Analyses focused on single gene associations (previous section) may overlook cases in which an upstream regulator affects a morphological trait through its influence on other genes. To investigate this possibility, we carried out a two block partial-least squares (2B-PLS) analysis [54]. This technique, which bears some similarity to PCA, is applied to two sets of data, in this case gene expression measures and morphological measures, and seeks to find weighted groupings within each dataset that together maximize the correlation between the two sets of traits. This multivariate correlation is quantified by the RV statistic [55], which is analogous to Pearson's r^2^.

Using 2B-PLS analysis, we found a modest but statistically significant overall relationship between variation in the expression of active genes and in skeletal morphology (RV = 0.163, *p* = 0.002; permutation). A large weight in this association (49%) was attributable to the first pair of factors. 84% of the weighting in the skeletal factor of this first pair was associated with the body rod length and thus with the overall length of larvae (Tables S6 and S7). Interestingly, the strongest associations showed a strikingly nonrandom distribution among developmental time points. The 10% most highly weighted genes in the first expression factor showed a strong enrichment (11 of 22 expression measures) for expression at time point 1 (*p* = 5.42×10^−7^, χ^2^ = 25.108, df = 1). A further seven of these top 10% expression measures corresponded to later stages of development (Table S8), including two associations (time points 5 and 6) with *SM30-E*, which encodes a major component of the skeletal matrix. Only a few expression measures (three of 22) among the most heavily weighted components of the first gene expression axis involved regulatory genes at intermediate time points: *Pmar*, which encodes a transcription factor critical in skeletogenic cell fate specification, and *SM50* and *SM27*, both of which encode components of the skeletal matrix with primary functions in later in development (Figure 6C).

Next, we examined the influence of gene expression on multidimensional variation in skeletal morphology by calculating the total contribution of each expression measure as the weighted sum of its loadings on each of the six 2B-PLS factors. The 11 gene-time point combinations that comprised the upper tail of the distribution (top 5% of scores) were again heavily weighted towards the top and bottom of the network (Figure 6D; Table S9). Six of the expression measures correspond to transcription factors expressed during the first time point, while two others involve *Pmar*, which was also associated with skeletal size (previous paragraph), at time points 2 and 3. Included in this list were only two genes expressed later in development: *SM30-E* (time points 5 and 6), a second gene that was also associated with skeletal size in our PCA-based analysis (previous paragraph), and *FoxO* (time point 6), which encodes a transcription factor that regulates the epithelial-to-mesenchymal transition that skeletogenic cells undergo prior to commencing biomineralization (L. Saunders and D. McClay, submitted).

Thus, the 2B-PLS analyses indicate that the strongest gene expression-morphology associations are concentrated bimodally, in very early development and in terminal differentiation.

### Variation in Gene Expression Influencing Skeletal Morphology Has a Non-maternal Genetic Component

Since skeletal variation and many of the genes uncovered by our correlation analyses are both influenced by maternal effects, the correlations we observed between them could be due to covariation with a common maternal influence. To test this possibility, we sought evidence of a statistically significant correlation between gene expression and a principal component of skeletal variation by including a maternal parental term as an additional factor in our linear models. For any gene-time point expression measures that remain significant predictors of skeletal variation even after accounting for maternal effects, we can reject the hypothesis that maternal effects are the sole factor influencing correlations between gene expression and skeletal variation.

Using this approach, we could not reject a model that included only maternal influences for the majority of genes within the network that showed some prior association with skeletal variation. However, for four genes (*FoxO*, *SM30-E*, *Msp130*, and *C-lectin*) we found significant support (*p*<0.01) for non-maternal co-variances at time points 4 and/or 5 (Figure 6E). The relationship between the expression of these four genes and skeletal morphology appears to be independent of maternal effects, and thus is most likely due to genetic contributions. Because our ability to detect maternal genetic contributions is confounded by non-genetic components of egg quality and is limited by the modest size of the cross, it is certainly possible that additional associations between gene expression and morphology have a genetic component.

## Discussion

The position of a gene within a regulatory network may determine the extent to which mutations altering its function influence trait variation and adaptation [7],[56]. One way to measure this impact and to understand why certain genes and not others underlie trait variation is to undertake a systematic analysis of the distribution of functional variation during development that contributes to the trait of interest. In this study, we assessed the impact of natural variation on both proximate (gene expression) and organismal (morphology) quantitative trait variation within the context of a well-defined developmental gene network (Figure 1). The results highlight the significance of a gene's position within the network, both for buffering the consequences of genetic variation during development and for providing adaptive modifications to development during the course of evolution.

### Natural Variation in Network Function Is Extensive and Has a Genetic Component

We observed extensive variation in gene expression within the gene regulatory network in a natural population. Most involves modest changes in the timing or level of gene expression, although two genes in particular (*Pmar* and *VEGF1R*) showed striking differences (Figure 2). Based on our NCII breeding design, we found evidence for a genetic component to expression variation for most genes (Table S1). The majority of these showed statistically significant paternal influences even during early development and despite the modest size of the cross, suggesting that genetic contributions to variation are often substantial. This result is consistent with the extensive genetic variation within regulatory elements (including transcription factor binding sites) that previous studies have reported for *S. purpuratus* [33]–[36]. Average levels of genetically based variation in gene expression remained appreciable throughout development, with a modest drop after the second time point (Figures 3 and S1). These results are consistent with a previous study of genetic contributions to gene expression during development [57], and extend them to natural populations and to a broad range of developmental stages.

Understanding the phenotypic consequences of variation in gene expression within wild populations is important for three reasons, which we discuss below: (1) much of it must be buffered so as to avoid adversely affecting later developmental processes, (2) it may influence quantitative variation in ecologically significant organismal traits such as morphology, and (3) it may form the basis for future adaptations.

### Developmental Buffering Impacts Network Function and Genetic Variation

An important question is whether the variation in gene expression encountered in nature is buffered or propagated across a gene network during development [2],[5],[58]. Consistent with what one would expect of transcriptional regulators and their targets, we found that covariation in gene expression was generally higher for direct regulatory interactions than for random pairs of genes within the network (Figure 4A). However, the levels of covariation we observed were generally modest or nonexistent, indicating that the effects of variation in gene expression are buffered throughout the network. One possible mechanism for buffering transcriptional regulation is through threshold, or switch-like, interactions. Switch-like regulation of target gene expression can arise in several ways, including cooperative binding of transcription factors [59]–[61] and transcription factor saturation at binding sites [62],[63]. In either case, changes in transcription factor concentrations above a certain threshold would have relatively little impact on the expression of their target genes. We found that many regulatory interactions in *S. purpuratus* show either switch-like behavior (e.g., Figure 5B) or a low degree of correlation, suggesting that transcriptional regulatory interactions can contribute to developmental buffering. Other regulatory interactions within the network, however, showed stronger correlations between regulator and target (e.g., Figure 5A). An important topic for future research will be understanding what makes these interactions different. Do they represent cases in which cooperative regulatory interactions play a less important role in regulating gene expression levels or where transcription factor binding sites are rarely saturated? The fact that less buffered interactions often lie upstream of terminal differentiation genes may point to an important difference in how genes are regulated during cell fate specification versus differentiation.

Our results indicate that variation in gene expression is buffered particularly well during early development. One manifestation of this is an overall increase in covariation between the expression levels of neighboring genes within the network as development proceeds; in early embryos, the expression levels of genes involved in direct regulatory interactions are no more correlated than those of random pairs of genes, while at later stages, expression levels of direct interactors are significantly more correlated than random pairs (Figure 4A). Another indication of a change in the degree of buffering during development is a substantial increase in the proportion of sensitive regulatory interactions over time, from about one-third during early embryogenesis to about two-thirds at later stages (Figure 4B).

These qualitative shifts in the nature of regulatory interactions may be related to changes in the overall function of the network during development. Early development involves a series of binary decisions between distinct cell fates. Buffering may be especially important during this time, to ensure the fidelity of all-or-nothing cell fate decisions and to allow for the proper integration of distinct sets of regulatory inputs. Buffering may also be important during early development to screen out environmental variation that might otherwise influence these critical processes. Consistent with this expectation, we previously reported minimal influences of ecologically relevant thermal stress on gene expression throughout this network [64]. Later development, in contrast, involves the inherently quantitative processes of growth and morphogenesis. Quantitative regulatory interactions among genes in a network may be especially important during post-embryonic stages in order to fine-tune these quantitative processes in response to nutrition, pathogens, physical conditions, and other environmental factors [65].

Note that buffering and cell fate specification are not necessarily related processes. Binary cell fate decisions may depend on a threshold response to a molecular cue, but that cue may be a property other than transcript abundance of a single upstream gene. Conversely, quantitative processes like growth could be based on the additive effects of many switch-like regulatory interactions of small effect. Nor is buffering a necessary component of cell fate specification, since there is no reason why a network with reduced variation in gene expression could not execute binary cell fate decisions.

Our results also suggest that developmental buffering can influence the accumulation of genetic variation that influences development in wild populations. We observed more genetically based variation in the expression of genes upstream of switch-like (insensitive) regulatory interactions than in those upstream of quantitative (sensitive) interactions. Our results further suggest that genetic variation in the natural population is not distributed randomly across the gene network, but is instead a by-product of the change in developmental mechanisms from cell fate specification in the early embryo to morphogenesis and growth during later stages. We hypothesize that by masking variation during early development, switch-like regulatory interactions may allow cryptic genetic variation to accumulate over evolutionary time at nodes that operate early within the network. Cryptic genetic variation is evolutionarily significant, because mutation or stress can unmask it, sometimes with dramatic phenotypic consequences [9],[66],[67]. Our results may also help to explain the observation that expression profiles are often less conserved between species during very early development than at subsequent stages [68], a pattern that has long been noted for morphology across developmental stages [69]–[72].

### Variation in Gene Expression Influences an Ecologically Significant Trait

The larval skeleton of sea urchins plays critical roles in feeding, defense, and orientation [26]–[28],[30], and natural variation in this structure has an impact on mortality [27],[29]. Thus, genetic variation that influences the size and shape of the larval skeleton will likely be a target of natural selection in the wild. We found that variation in the expression of a few genes within the network is associated with quantitative variation in the morphology of the larval skeleton (Figure 6). These genes fall into two sets: one expressed during early embryogenesis and the other during skeletogenesis.

Correlations between the first set of genes and skeletal morphology are unlikely to represent a causal relationship that is mediated through transcription of the network genes we examined. Support for this inference comes from the lack of correlation between the expression of genes in the middle portion of the network (Figure 6C–6F) and skeletal variation: if early gene expression influenced the skeleton through the network, the expression of intermediate genes should also be significant in the 2B-PLS analysis. Another reason is that the association between early genes and skeletal variation is fully explained by the maternal parent. Thus, it seems likely that a single maternal input, such as egg size or provisioning, independently influences variation in the expression of early genes and variation in larval morphology. The existence of such an input would explain why expression levels, as well as breeding values, are relatively highly correlated at time point 1 (Figures S2 and S3; Text S1) and is supported by experiments demonstrating that variation in egg size and quality influence the morphology of the larval skeleton [27],[47],[48]. Nonetheless, we cannot rule out the possibility that these genes act through the network via processes other than transcription or through interactions outside of this network such as changes in cell size.

In contrast, the second set of genes are likely candidates for directly contributing to variation in the larval skeleton. All are expressed during formation of the skeleton, most are expressed exclusively within skeletogenic cells, and most encode protein components of the biomineral matrix itself. That many of these genes change expression in response to targeted chemical manipulations that alter of the size and structure of the larval skeleton [73]–[75] and that they covary with skeletal morphology independent of maternal effects (Figure 6) provides further support for a direct connection between their expression and skeletal formation. These results are consistent with the idea that the position of a gene within the network has consequences for quantitative genetics and that mutations affecting the expression of terminal structural genes are among the most likely to influence variation in the size and shape of the larval skeleton. Importantly, this hypothesis can be tested directly by manipulating gene expression levels experimentally and measuring trait consequences.

Terminal genes may influence morphological variation simply because fewer molecular events separate them from the trait of interest than is the case for genes that operate farther upstream, providing less opportunity for buffering. Other studies have also found that the expression of terminal genes can influence an organismal trait, as with abdominal pigmentation in Drosophila [10],[76],[77]. It is important to bear in mind, however, that terminal genes are not always major contributors to phenotypic variation. In metabolic networks, variation in genes at the top of the network often have the largest impact [78]–[80], while in developmental networks, genes at intermediate positions sometimes contribute the most to trait variation [37],[38],[81]. A clue as to why these cases differ may lie in the nature of gene interactions. Most of the interactions that regulate the structural genes of the sea urchin larval skeleton are quantitative rather than switch-like, which allows variation in immediate upstream regulators to influence the expression levels of structural genes, whereas changes in more upstream genes are buffered.

In light of the number of regulatory interactions that appear to be buffered in this network, associations between genes encoding transcription factors such as *FoxO* and skeletal morphology become all the more surprising. Unlike structural genes such as *SM30-E*, the impacts of changes in transcription factor expression are necessarily indirect, and it is worth considering why some transcription factors, but not others, quantitatively influence morphology. Differences in molecular mechanisms of action may provide important clues. For instance, forkhead proteins, including FoxO, often act as pioneer transcription factors, binding directly to condensed chromatin and setting the stage for lineage-specific transcription by recruiting chromatin remodelers and additional transcription factors [82]–[84]. Forkhead transcription factors have also been proposed to play a special role in fine-tuning gene expression [85]. Although the regulatory targets of FoxO remain unknown in sea urchins, it is tempting to speculate that the quantitative association between FoxO expression and skeletal morphology stems from a potentially rate-limiting role in priming the chromatin landscape for transcription. More generally, this result highlights the need to better understand the molecular mechanisms that link genes with the organismal phenotypes that they influence.

### Expression Variation in Terminal Genes Provides Raw Materials for Adaptation

Several elegant studies of adaptation in wild populations have traced ecologically important phenotypes to changes in the transcriptional regulation of a single gene during development [10],[12],[38],[86]. Moving forward, a central challenge in evolutionary genetics is understanding why certain genes, and not others, contribute to adaptation. Systematically measuring variation and correlations across a gene network and across developmental time, as we have done here, provides one promising approach.

For a gene to contribute to adaptation, it must harbor variants that influence its function, and this variation must be associated with an ecologically significant organismal trait. Based on these criteria, *C-lectin*, *FoxO*, and *SM30-E* are the strongest candidates among the genes we examined for contributing to adaptive changes in the size and shape of the larval skeleton in *S. purpuratus*. Additional candidates are *FoxB*, *Msp130*, and *SM50*, though with somewhat weaker support. Most of the remaining genes we assayed show no clear correlation with skeletal morphology and seem less likely to contribute. Five of the six candidates are terminal differentiation genes and the other is a transcription factor expressed during differentiation, suggesting that network position is a significant factor in the genetics of adaptation for the larval skeleton.

Several independent lines of evidence suggest that the larval skeleton of *S. purpuratus* should be able to evolve adaptively. First, this species has an enormous effective population size, non-assortative mating, and extensive gene flow [32],[87],[88], all features that favor the efficient operation of natural selection. Second, populations of *S. purpuratus* harbor substantial genetic variation (0.5%–3%) in noncoding regions of the genome [31],[36],[89], providing abundant genetic variation upon which selection can act. This includes extensive genetic variation within empirically validated cis-regulatory elements and transcription factor binding sites [33]–[36]. Third, evidence presented in this study indicates that some of this genetic variation has an impact on phenotypic variation in the size and shape of the larval skeleton.

Adaptation requires a fourth component that is more difficult to assess, namely the ecological circumstances that favor phenotypic change. As mentioned earlier, the skeleton plays several important roles in larval feeding, defense, buoyancy, and swimming, making it a likely target of selection [26],[28]–[30]. The remarkable diversity of skeletal size, shape, and configuration among living species of sea urchins points to extensive adaptive changes over the past 250 million years of evolution [72],[90], while signatures of positive selection within the cis-regulatory elements of genes in the skeletogenic network indicate recent adaptive changes within *S. purpuratus* [34]–[36]. Thus, adaptation in skeletal size and shape has likely been an important part of the evolutionary history of sea urchins, and the variation we document here has the potential to contribute to future adaptation.

### Network Position Has an Impact on Trait Variation and Adaptation

Our results reinforce the idea that the position of a gene within a regulatory network position has important evolutionary consequences [6]. Some regulatory interactions within the developmental network of sea urchins can buffer more variation than others, and the distribution of these interactions is enriched during embryogenesis relative to later development. As a consequence, the accumulation of genetic variation affecting developmental mechanisms within the population is likely to be nonrandom across the network and across developmental time. We also found that genetically based variation in the expression of a subset of genes is correlated with variation in an ecologically relevant trait, the larval skeleton. This variation is also not randomly distributed across the network, but enriched in genes that encode structural proteins and that have cell type-specific expression. This suggests that the genetic basis for adaptation in the larval skeleton is likely to come primarily from genes with terminal positions in the network. Empirical investigation of well characterized gene regulatory networks may eventually allow one to formulate predictions about the genetics of adaptation.

## Materials and Methods

### Cross Design, Rearing, and Sampling

The adult sea urchins used for the cross were collected during a single SCUBA dive from a population in the Santa Barbara Channel, Santa Barbara, California (US). Individuals were shipped overnight to Durham, North Carolina and held in aquaria containing artificial sea water (Coralife, Oceanic Systems Inc.) at 12°C for <48 h prior to spawning. Gametes were obtained from similarly sized individuals (a proxy for similar age) and fertilization was carried out following standard procedures [91]. With the resulting gametes, we set up a North Carolina II breeding design [41],[92] consisting of all pair-wise crosses between six male and six female urchins. Each culture was raised in a randomized design in artificial sea water at 12°C in controlled climate chambers at the Duke Phytotron (Durham, North Carolina). Temperature was monitored continuously using sensors distributed throughout the growth chambers. Because we made use of a randomized design, neither micro-environmental variation nor sampling order should influence our estimates of parent-of-origin effects (or additive genetic variation). Uncontrolled variation did, however, introduce phenotypic variation across the experiment that improved our ability to detect patterns of correlation between pairs of genes at each time point.

The 36 pools of zygotes generated by our cross were reared in replicate, and all 72 cultures were sampled at seven developmental time points: 10, 18, 24, 28, 38, 45, and 90 h post-fertilization. These time points span very early embryogenesis through a free-swimming larva capable of feeding (Figure 1A). All 72 cultures came from crosses that yielded at least 95% fertilization rates, but 22 cultures had somewhat higher rates of malformed embryos, as assessed by no or irregular early cell divisions. We excluded these cultures from analyses at time points 1 and 2 because we could not reliably separate healthy from malformed embryos. However, we were able to include data from these cultures in our analyses for time points 3 to 7 by taking advantage of the fact that healthy embryos begin swimming between time points 2 and 3, allowing a clean physical separation of healthy from malformed embryos. A minimum of several hundred embryos were sampled from each culture at each time point for RNA extractions, with numbers varying somewhat owing to changes in total RNA per embryo during development.

### RNA Extraction and Gene Expression Measurements

RNA extractions were carried out using the Qiagen RNeasy 96 kit (Qiagen), quantified on a NanoDrop (Thermo Scientific), and adjusted to between 10 and 100 ng/µl with water. RNA integrity was checked in ten samples using an Agilent 2100 Bioanalyzer. None of the samples showed evidence of RNA degradation. Genomic DNA extractions were carried out using the Qiagen DNeasy mini kit (Qiagen), DNA quantity measured on a NanoDrop, and adjusted to between 20 and 100 ng/µl with buffer AE.

We used Illumina's DASL platform (cDNA-mediated annealing, selection, extension, and ligation) [93] to measure gene expression from these samples. This assay is based on the technology in the widely used GoldenGate genotyping platform and is well suited to measuring the expression of a moderate number of genes in a large number of samples. Briefly, fluorescent probes complementary to the targets of interest are bound to beads etched with a barcode. These probes are then annealed to cDNA in solution and captured in a plate. The expression levels of individual beads is read using a laser that simultaneously captures information about the quantity of cDNA bound to any bead as well as the barcode of that bead, thus identifying the identity of the fluorescent transcripts being interrogated. On average, ∼30 individual beads are measured per probe per sample.

We worked with Illumina to design a custom DASL assay containing 384 probes that targeted exons of 77 genes, based on annotations from SpBase [15] mapped to sea urchin build 2.1 (www.spbase.org). Where possible, we validated sequences from the sea urchin genome sequence against targeted sequencing efforts available in GenBank. We chose three to six probes with Illumina final scores >0.8 (App version 6.4.1.0.0.0:2.0.0) for each gene. Illumina recommends using three probes per gene to improve measurement precision. We included more probes when possible so that we could identify poorly performing probes on the basis of the correlations of all probes targeting the same transcript. The full set of probe sequences and the genes they target are available upon request.

DASL assays were carried out on the Illumina BeadStation by the Duke Genotyping Core Facility at the Duke Institute for Genome Sciences & Policy. RNA and gDNA samples, used for quality control, were processed and run separately. Raw bead-level data was recorded directly instead of passing through the Illumina BeadStudio software. At each stage of development, gene expression measures were normalized to the expression of RBM8A following correction for background fluorescence (Text S1). Note that, as with all methods for measuring transcript abundance, measurement error on the DASL platform is generally higher when expression levels are very low. However, relatively few genes in our set are expressed at very low levels during the developmental stages when they are known to participate in regulatory interactions.

### Scaling Gene Expression Measurements

To facilitate comparisons among genes, we normalized trait variances by the square of the mean expression of each gene over all cultures. The square root of this quantity is thus the coefficient of variation, an easily interpretable quantity in terms of percent change relative to the mean, and is recommended for comparing variances among traits [94]. An advantage of our network is that gene activity is defined both by expression and functional experiments. The coefficient of variation thus represents variation around a mean level of expression sufficient for gene function and is, thus, likely a more accurate predictor of the impact of variation in expression than an un-scaled measure of a gene's variance (i.e., some genes are naturally higher expressed than others, and a variance of one unit is likely more important for a gene expressed at only a few copies per embryo, than for a gene expressed at a very high level).

### Developmental Gene Regulatory Network Curation

In our analyses of this gene regulatory network, we benefitted from extensive prior work by other labs, much of which has been compiled into a Biotapestry [95] database available at (http://sugp.caltech.edu/endomes/; Figure 1B). From this database we extracted an XML representation of the network (version: 12 May 2009) and parsed the resulting information using custom scripts. In the XML data, time series information is recorded as gene activity reports every 3 h from 6–30 h post-fertilization. Since we reared our culture at a different temperature than that of the Biotapestry representation (12°C rather than 15°C), we converted times using published developmental schedules [91]. Edges between genes are also recorded along with the tissues in which the interaction occurs and the times over which the interaction occurs. At each developmental stage, genes were determined to be “active,” (rather than “expressed”) if they are expressed at detectable levels and take place in one of the regulatory interactions noted in this database (Table S10). Several nodes in the graphic representation of the network involve interactions between multiple proteins. In deciding which gene expression correlations would represent this node, we deferred to the edge representations in the XML data (e.g., the proteins TCF and β-catenin often work together to active transcription, but only β-catenin is listed in the XML representation). Genes that act as their own regulators were not included in subsequent analyses as we were not considering between time-point correlations.

In order to design probes for the DASL assay, we needed to assign each gene represented in the network to an annotated gene in the sea urchin genome project (SpBase.org) [15]. In the vast majority of cases, the assignment was either intuitive or could be quickly determined by matching probes, primers, or DNA sequence from prior publications to the sea urchin gene models. One exception was the node annotated as “*FvMo1,2,3*,” which represents three members of the flavin-containing monooxygenase gene family that are co-expressed in developing pigment cells and are strongly suspected to be co-regulated [96]. Unfortunately, it was not possible to unambiguously assign these genes to the current sea urchin genome assembly. We thus chose to use the well annotated *SpFmo2* gene as a proxy for the activity of this cluster both because it had been used as a proxy in recent analyses of the network [97] and because its putative cis-regulatory region contains likely binding sites for both annotated upstream regulators of the cluster (*Gcm* and *GataE*) [98].

### Quantifying Skeletal Variation

We measured the size and shape of the larval skeleton at time point 7 (90 h post-fertilization) by marking eight established morphological landmarks in three dimensions [99] on images from a dense z-stack taken at 3 µm intervals and used these landmarks to define distances corresponding to lengths of the skeletal rods that make up the larval skeleton. Images were taken of 18–30 larvae per culture, measured using ImageJ, and rod lengths calculated using a custom Python script. We took two approaches to examining the relationship between gene expression variation and variation in the larval skeleton. First, we used PCA to collapse our measures to three factors, which capture 88% of the between-culture variation in skeletal rod lengths and show strong parent-of-origin (particularly maternal) effects (Text S1). We then measured correlations between each factor and the expression of each gene at each time point (Table S5). For the second approach, we carried out a two block partial least-squares analysis [54],[55] to test for components of the gene expression variation and skeletal variation data that together maximized the correlation between the two datasets. See Text S1 for further details of both approaches.

### Classification of Regulatory Interactions

Correlations between the expression levels of interacting genes vary both quantitatively and qualitatively (e.g., Figure 5). To better understand how these patterns of correlation change over development, we employed two methods. First, we asked if r^2^ values between directly interacting genes were, on average, stronger than those between active genes with no known regulatory interactions. To address this question, we compared the average r^2^ values of all interacting genes at each time point to the average of all non-interacting genes. To test for statistical significance, we compared the observed results to 10,000 permutations of the data by randomizing the edges but keeping the overall network topology intact. With the second method, we asked a slightly different question: How does the qualitative nature of regulatory interactions change over development? We classified each edge (regulatory interaction) in our curated network representation into one of two types: (1) Switch-like or “insensitive” interactions, in which a downstream gene is insensitive to quantitative variation in the upstream gene, and (2) “sensitive” interactions, in which there is a statistically significant (*p*<0.01) relationship between variation in an upstream gene and its downstream targets as assessed by standard linear regression. Both sets of analyses were conducted with and without repressive regulatory interactions with no impact on the statistical significance of the results.

### Differences in Tissue Composition among Broods

Differences in tissue composition among broods could affect gene expression variation and increase the apparent correlations in expression values for genes expressed in the same tissue. To test for this possibility, we made use of the fact that most of the genes analyzed in our study are classified into one (or more) of nine non-overlapping domains/tissues of expression at each time point in the Biotapestry database (covering time points 1–6) from which we extracted information about network topology. Figure S2 shows all pairwise correlations for each gene marked as expressed within a tissue (plus all tissues in the upper right). The large number of negative correlations observed within tissues argues that changes in tissue composition are not a driving factor underlying gene expression correlations. This observation is supported by formal statistical analyses: at no time point are correlations within a tissue greater than between random genes (all *p*>0.2, 1,000 permutations), a result that holds when the analysis is restricted to genes expressed in one, and only one, tissue per time point. For thoroughness, we also conducted this analysis using correlations among breeding values (Figure S3) with similar results (both qualitatively and formally).

### Estimates of Parental Effects and Genetic Variances

A requirement for the operation of natural selection is the presence of additive genetic variance, that is genetic variation that has a significant average effect on a phenotype across a range of environments and genetic backgrounds. One metric of additive genetic variance is four times the paternal or maternal covariance among half-sibs, namely individuals that share one parent, but not the other. In this experiment, estimates of these parental contributions were estimated using a North Carolina II breeding design using six outbred male and female *S. purpuratus* individuals of approximately the same age as described more fully in Text S1.

Parental effects in the NCII design are typically estimated using ANOVA methods. However, ANOVA methods are not well suited for estimating error terms or significance in the face of missing data. When analyzing gene expression data, we therefore converted the standard mixed-effect linear model underlying the NCII design into a Bayesian hierarchical mixed-effect model by adding priors on the genetic and residual variances and fitting the model using a Gibbs sampler, implemented in the MCMCglmm package in R [100]. We took this approach for two reasons. First, due to filtering for quality control of the gene expression measures, certain samples with low quality expression measurements were removed, resulting in an unbalanced design. Likelihood-based methods, such as REML and Gibbs samplers inherently tolerate unbalanced designs, while ANOVA methods require complicated adjustments [41]. Second, since our sample sizes were small for estimating variances, asymptotic closed form confidence-interval estimates on variance components such as those produced from REML or ANOVA methods, are not reasonable, and return confidence intervals that span zero for low variance estimates. Bayesian credible intervals can be much more interpretable in these situations as they do not depend on asymptotic assumptions and can be enforced to be positive and asymmetric. In our analyses, we used a diffuse inverse gamma prior centered at 10% of the expression of each gene for each variance term (see Text S1 for fuller discussion). If fewer than 20 samples had expression levels above background for a given gene at a given time point, no variance measures were calculated. The significance of specific linear models was calculated using DIC-based model comparisons, as discussed more fully in the Supporting Information. To confirm the validity of our approach, we also conducted more traditional REML-based analyses (Text S1). The two sets of results were highly concordant.

With an NCII design, it is theoretically possible to estimate the genetic covariances between genes. Owing to the relatively small size of our breeding design, however, we cannot accurately estimate genetic covariances between individual pairs of genes. We can, however, make attempts to compare the full set of genetic covariances (the G-matrix) between time points. One measure of genetic constraints in this case is the variance of the eigenvalues for the G-matrix at each time point: high variances indicate that the G-matrix is constrained primarily in one or a few directions, while low variance (all eigenvalues having similar magnitudes) is indicative of a relative lack of constraint. Via permutations tests, we can see that the G-matrices at each time point are more structured than random (Text S1), as one would expect from a network of interacting genes. However, only at time point 1 is the variance among eigenvalues statistically distinguishable from (and larger than) other time points, highlighting the greater extent to which genes at time point 1 (including those with no known interactions) share a common genetic influence (Text S1).

### Data Access

Data are available from the Dryad Digital Repository [101].

## Supporting Information

Figure S1 (A) Mean levels of additive genetic variation [2×(male + female effects)] at each developmental time point (log scale). Time points 1 and 2 show a slightly increased level of additive genetic variation relative to later time points that is highly significant (Wilcoxon, *p* = 3.48×10^−8^). The difference remains significant under a number of measures of additive genetic variation (see Text S1). (B) Mean log parental effects at each of the seven time points. Parental means for each gene were estimated with the mean of the posterior distributions in our Bayesian model (see Text S1).
(EPS)

Figure S2 **Edge correlations by tissue by time.** Plotted are histograms of the observed pairwise correlations between all genes expressed in each of the nine tissues as annotated in the Biotapestry database. Each page represents a different time point (1–6) with the correlations over all annotated genes plotted in the upper right. The blue bar notes 0 and the green bar the mean pairwise correlation in that tissue. If fewer than two genes were expressed in a tissue at a time point, the histogram is blank with a single green bar at zero. Following conventions in the Biotapestry database, abbreviations for regions are: Abo, aboral ectoderm; E, endoderm; EC, ectoderm; EM, endomesoderm; M, mesoderm; MAT, maternal; OE, oral ectoderm; P, primary mesenchyme/skeletogenic cell lineage; VE, vegetal. Numbers following these abbreviations refer to time points 1–6 of the present study.
(PDF)

Figure S3 **Breeding value correlations by tissue by time.** Plotted are histograms of the observed pairwise correlations between breeding values for genes expressed in each of the nine tissues as annotated in the Biotapestry database. Each page represents a different time point (1–6) with the correlations over all annotated genes plotted in the upper right. The blue bar notes 0 and the green bar the average pairwise correlation in that tissue. Following conventions in the Biotapestry database; abbreviations for regions are: Abo, aboral ectoderm; E, endoderm; EC, ectoderm; EM, endomesoderm; M, mesoderm; MAT, maternal; OE, oral ectoderm; P, primary mesenchyme/skeletogenic cell lineage; VE, vegetal. Numbers following these abbreviations refer to time points 1–6 of the present study.
(PDF)

Figure S4 **Morphological landmarks.** Pluteus larva (∼90 h post-fertilization) with the larval skeleton visible. Red dots represent standard morphological landmarks [46].
(EPS)

Figure S5 **Examples of bead masks.** These were used to control for spatial artifacts in the raw bead data.
(EPS)

Figure S6 **Distribution of number of beads used.** Each expression measurement in each sample is generated from the average expression estimated by a number of beads attached to the same probe.
(EPS)

Figure S7 **Example MA plots.** These show the dye-specific biases associated with the two dyes used to measure gene expression.
(EPS)

Figure S8 **Corrections to the distribution of the intensities of the different background-control beads.** (A) The distribution of background intensities by control bead before correction. (B) The distribution of background intensities by control bead after correction.
(EPS)

Figure S9 **The distribution of “expression” levels for DASL measurements applied to genomic DNA.** For details on how certain probes were removed on the basis of the divergence of their expression.
(EPS)

Table S1 **Single gene estimates of parental effects.** For each gene at each time point, summary statistics are provided for the output of our generalized linear model. This includes the mean, mode, median, and 90% credible intervals for male, female, interaction, and residuals, as well as DIC scores for each effect. The columns ‘male.sig’, ‘female.sig’, and ‘male.female.sig’ represent, respectively, whether or not the male, female, or interaction effects were significantly greater than zero based on permutations when using a REML-based approach (ASReml) for estimating parental effects. The columns ‘mean’ and ‘var’ list the unscaled mean and variance for each gene at each time point.
(TXT)

Table S2 **Summary of a linear model describing the relationship between variance and time point.** The intercept is forced to 0,0. As a result, the estimates are the mean of the total variance at each time point. Importantly, there is no relationship between variance levels and the fraction of sensitive edges described in Figure 4B.
(DOC)

Table S3 **The relative weightings of each skeletal measure in the first three principle components of skeletal variation.** These three axes explain 55.1%, 20.4%, and 13.4% of the total between culture variation in skeletal morphology.
(DOC)

Table S4 **Male, female, and interaction contributions to between family variation in the principle components of skeletal variation.** ** indicates *p*<0.01 using a standard likelihood ratio test.
(DOC)

Table S5 **Single gene correlations with morphology.** This table provides the full set of correlations between each gene at each time point and the first three principal components of larval skeletal variation, as measured at time point 7.
(TXT)

Table S6 **The weights of each skeletal measure's contribution to the six vectors summarizing skeletal variation produced by the two block partial least-squares analysis.**
(DOC)

Table S7 **Each of the six pairs of vector found by the two block partial least-squares analysis contributes different amounts to the total correlation between gene expression and skeletal variation.** The relative weighting of each of the six pairs of vectors are described by the eigenvalues given in this table.
(DOC)

Table S8 **The top 10% of gene expression contributions to the first 2B-PLS axis.** The column “Gene_Cluster_Time” gives the gene name followed by the cluster number (see Text S1 – “Gene expression DASL processing”) followed by the time point at which the expression measurements were taken.
(DOC)

Table S9 **By combining the gene expression contributions to each of the six axes weighted by the eigenvalue corresponding to each axis, we get a measure of the overall contribution of each gene_time expression to the overall relationship between gene expression and skeletal variation.** Above are the top 5% of total the weighted contributions of expression measurements to the overall correlation between gene expression and skeletal variation.
(DOC)

Table S10 **Edge activity.** This table describes each edge extracted from the Biotapestry database, the times at which the interaction occurs, the region in which it takes place, and whether the interaction is activating or repressive. The times correspond to the time points shown in Figure 1A. Note: only genes shown in Figure 1B were considered in this study.
(TXT)

Text S1 **Supplemental methods.**
(PDF)

## Abbreviations

2B-PLS: two block partial-least squares
PCA: principal components analysis
