# Supplementary figures and images for: The Impact of Gene Expression Variation on the Robustness and Evolvability of a Developmental Gene Regulatory Network
Source: PLoS Biol. 2013 Oct 29;11(10):e1001696. doi: 10.1371/journal.pbio.1001696 (PMC3812118; doi:10.1371/journal.pbio.1001696)

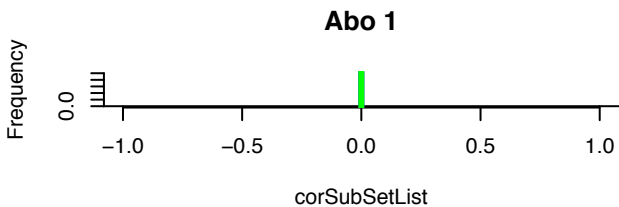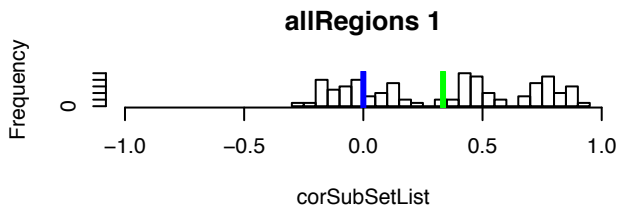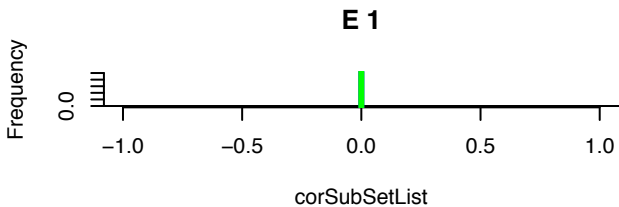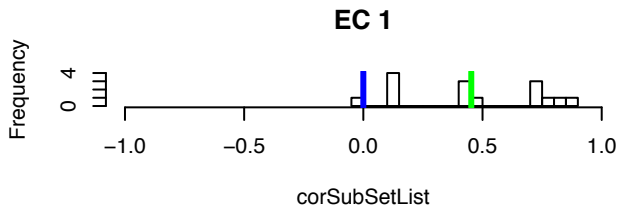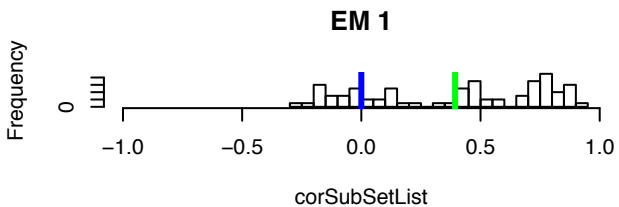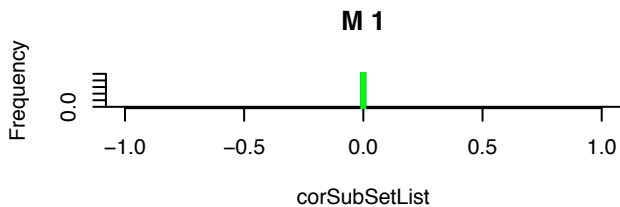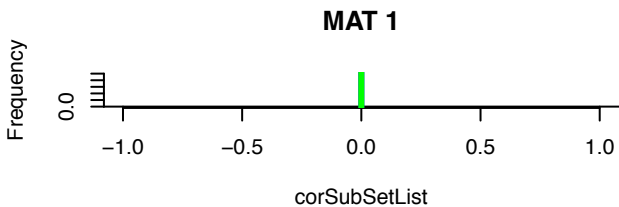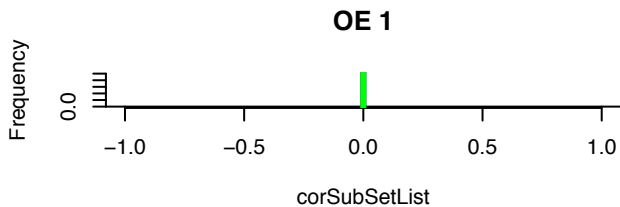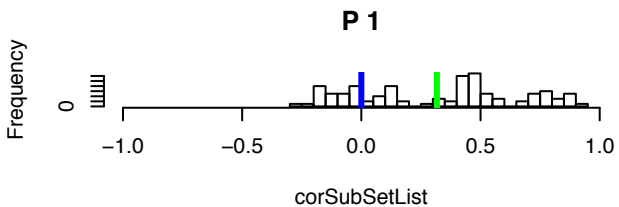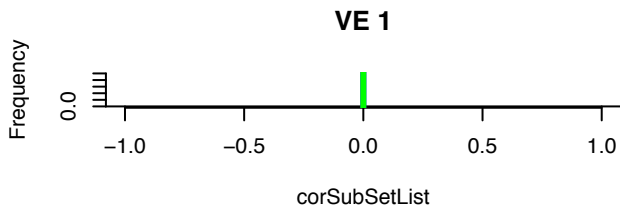

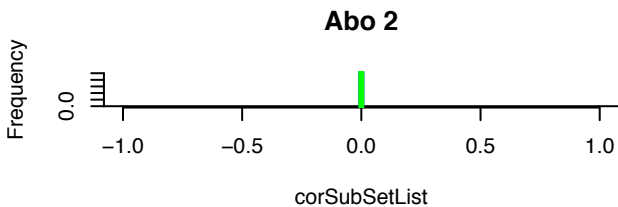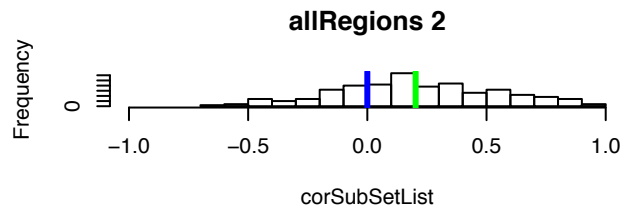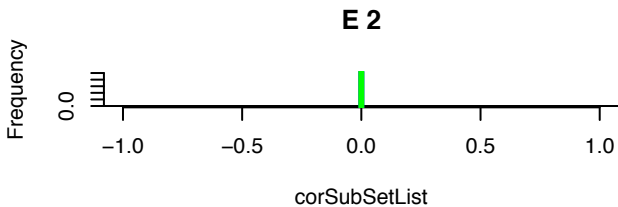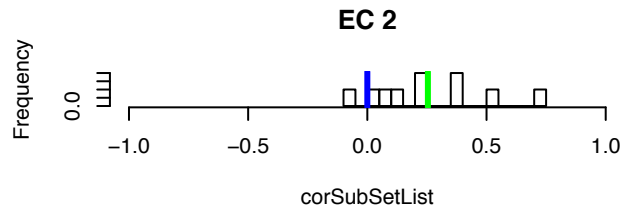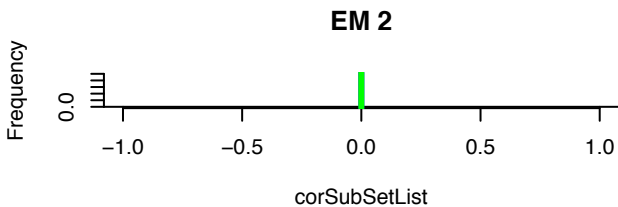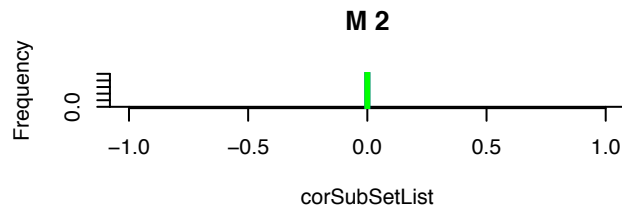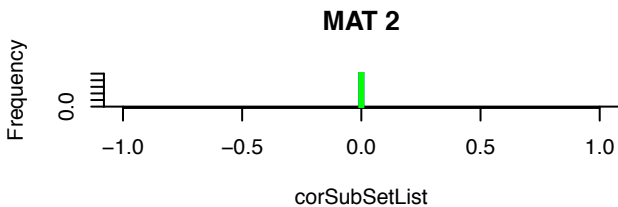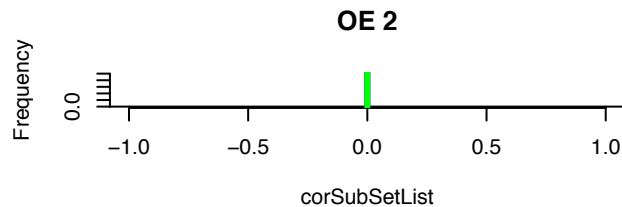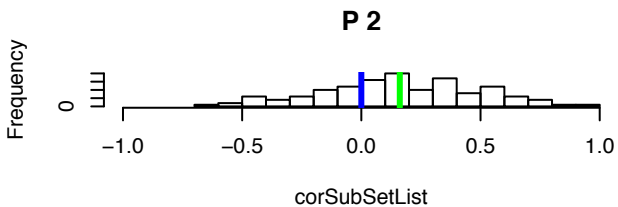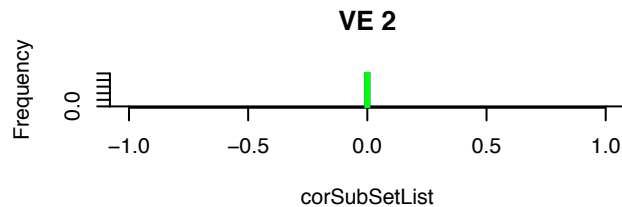

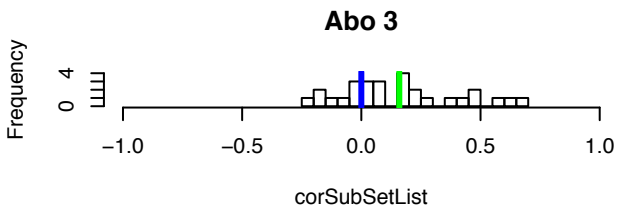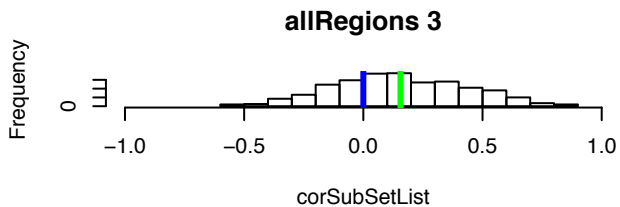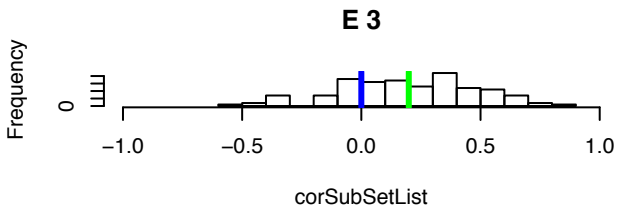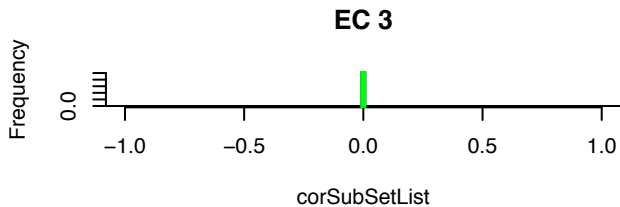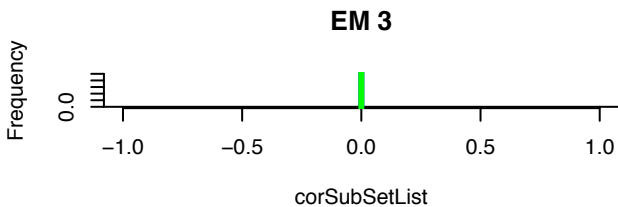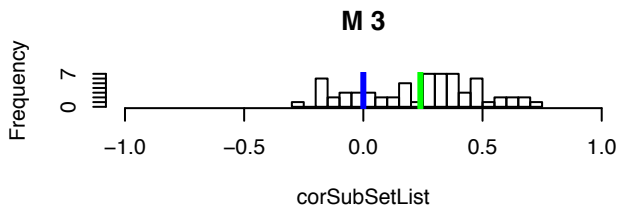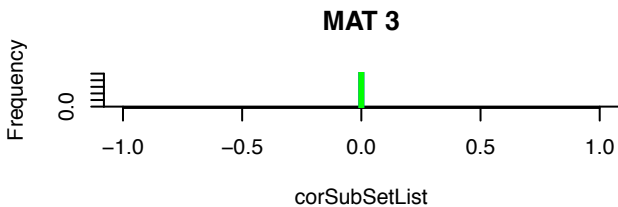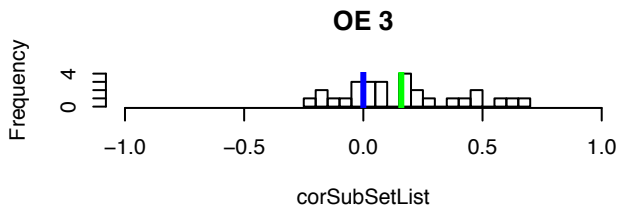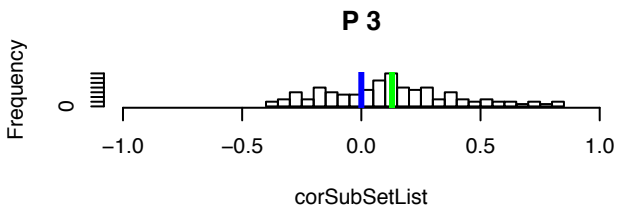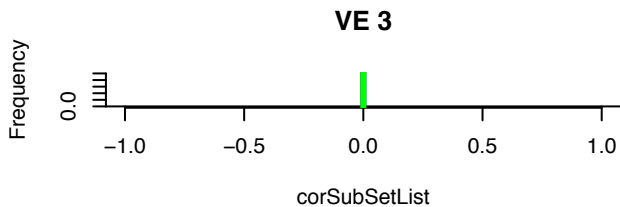

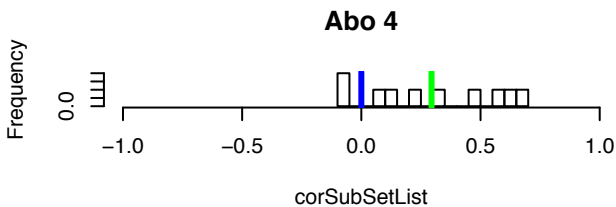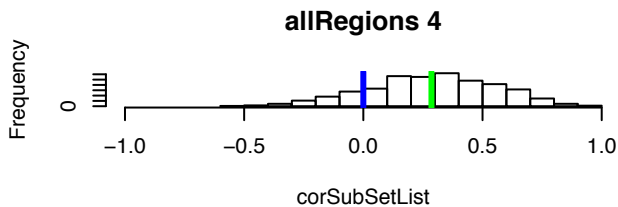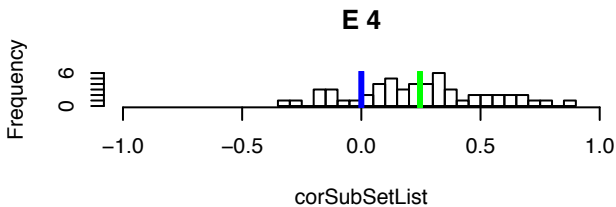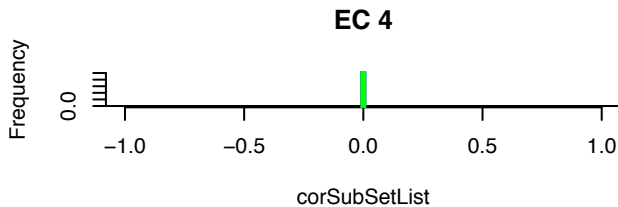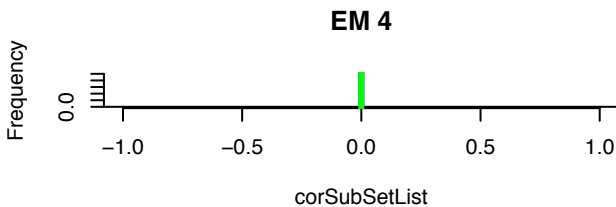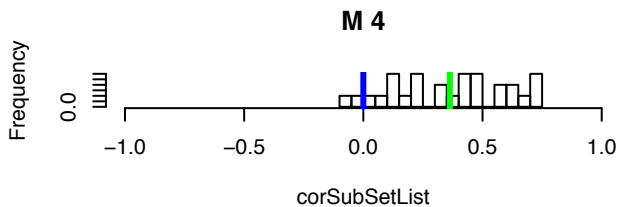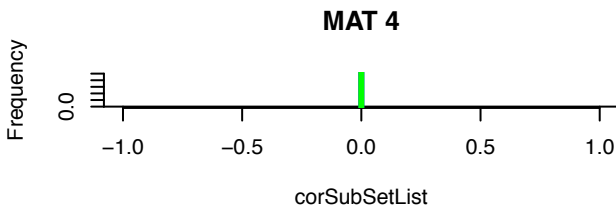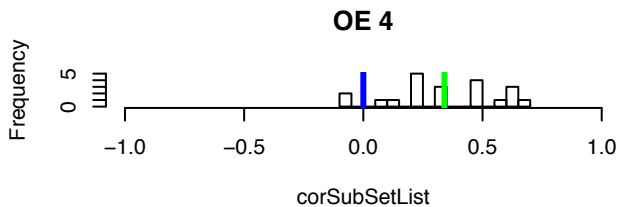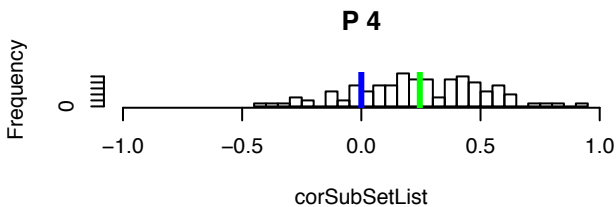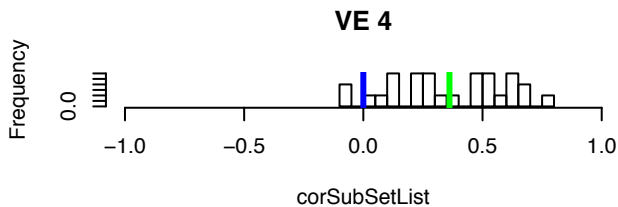

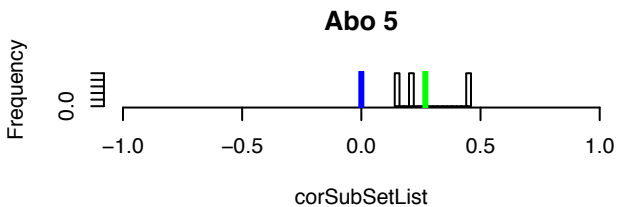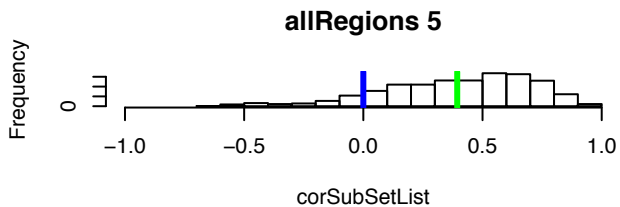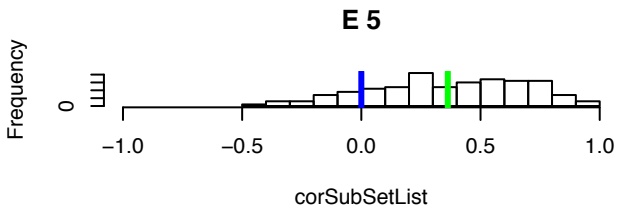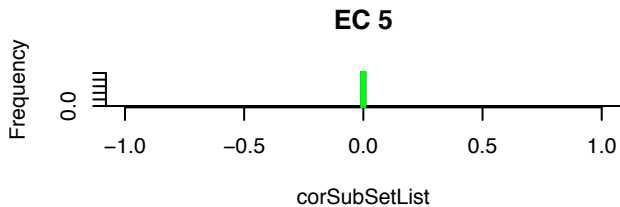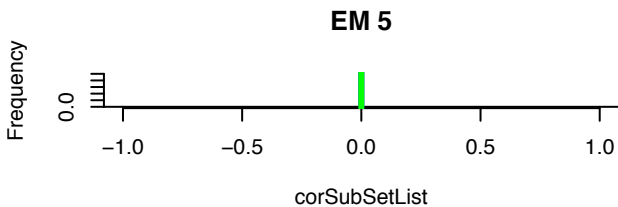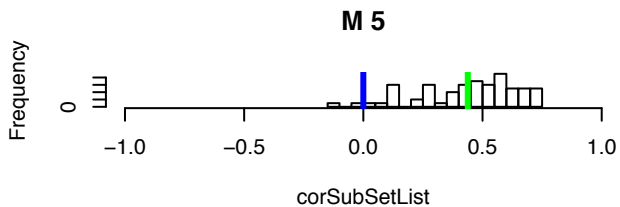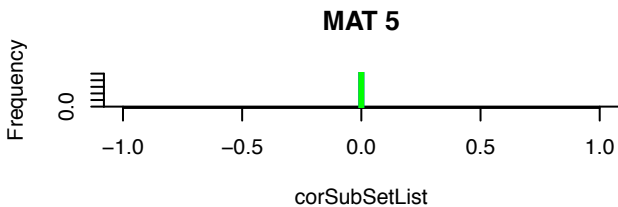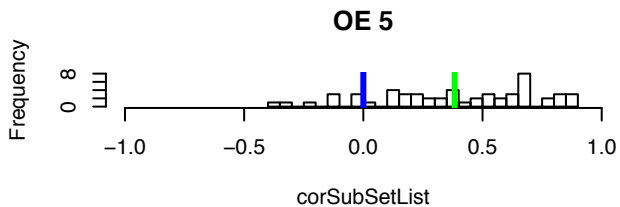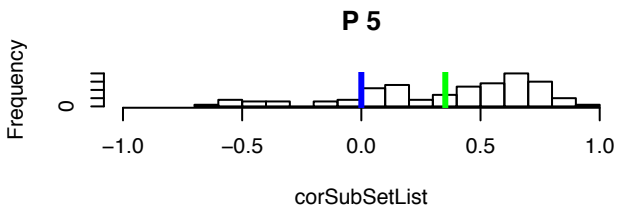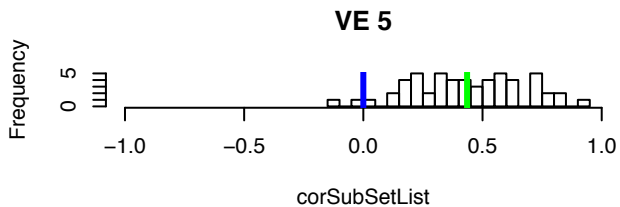

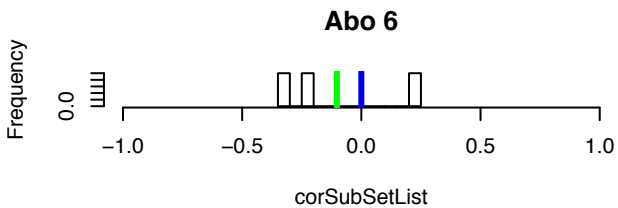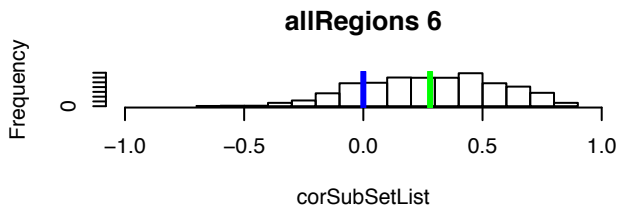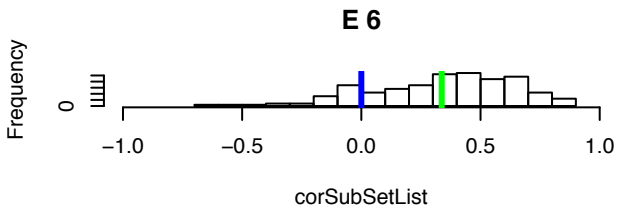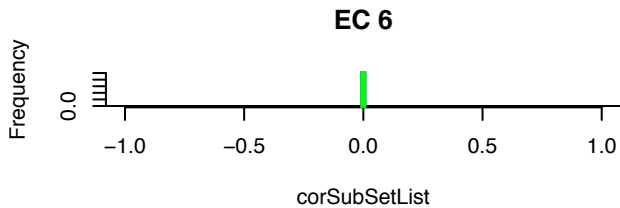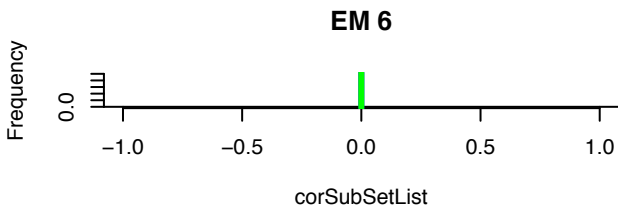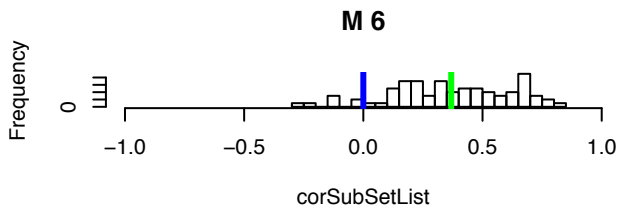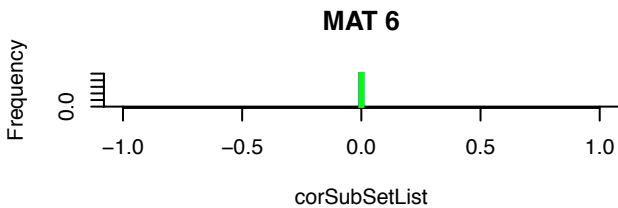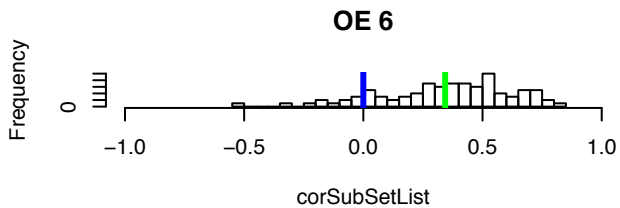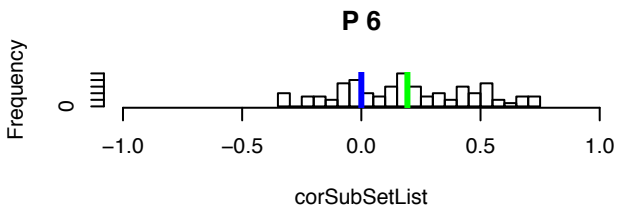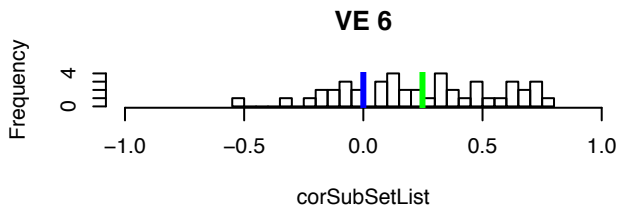

Supplement: Figure S2 — Edge correlations by tissue by time. Plotted are histograms of the observed pairwise correlations between all genes expressed in each of the nine tissues as annotated in the Biotapestry database. Each page represents a different time point (1–6) with the correlations over all annotated genes plotted in the upper right. The blue bar notes 0 and the green bar the mean pairwise correlation in that tissue. If fewer than two genes were expressed in a tissue at a time point, the histogram is blank with a single green bar at zero. Following conventions in the Biotapestry database, abbreviations for regions are: Abo, aboral ectoderm; E, endoderm; EC, ectoderm; EM, endomesoderm; M, mesoderm; MAT, maternal; OE, oral ectoderm; P, primary mesenchyme/skeletogenic cell lineage; VE, vegetal. Numbers following these abbreviations refer to time points 1–6 of the present study. (PDF) [file pbio.1001696.s002.pdf]

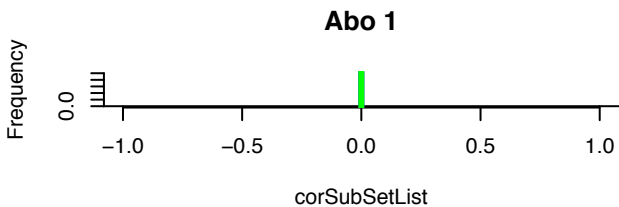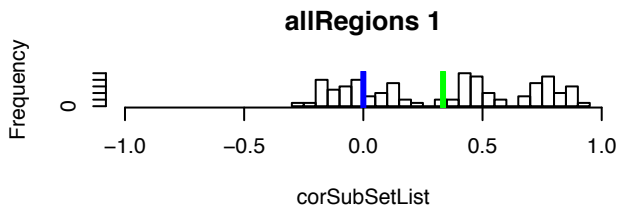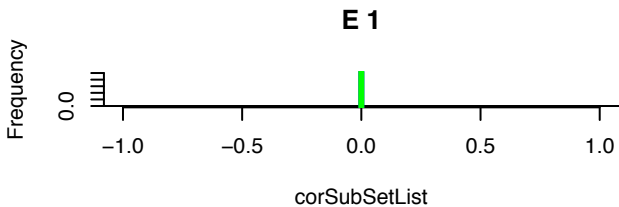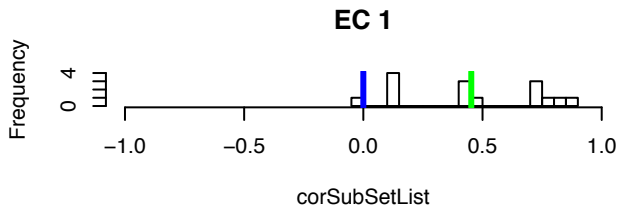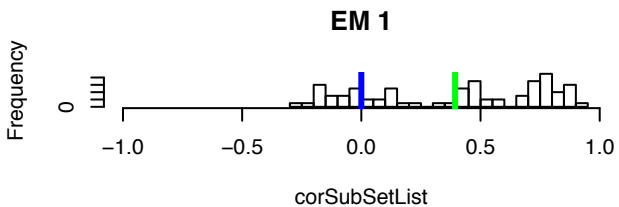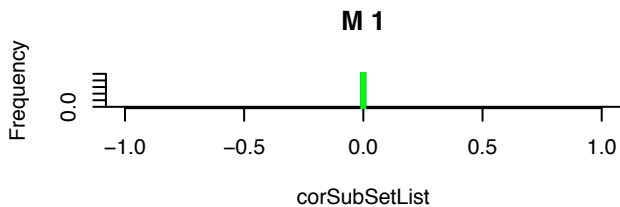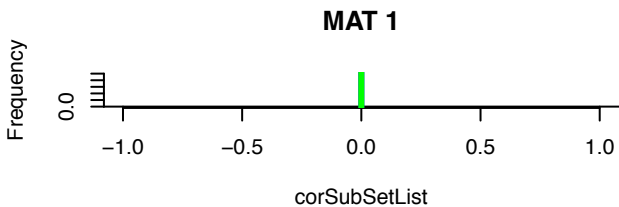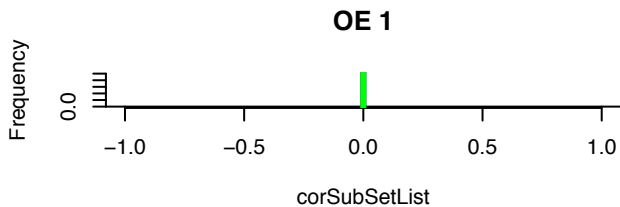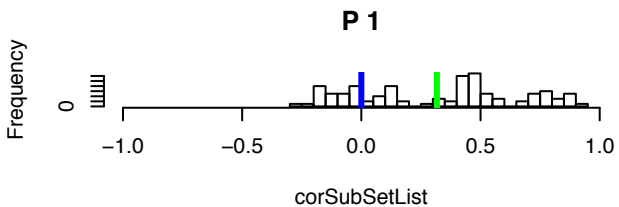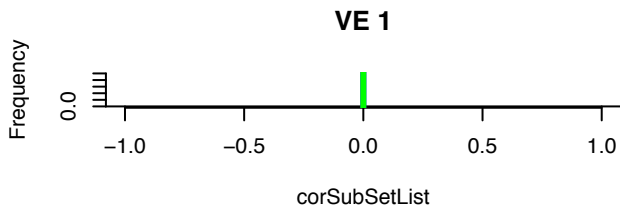

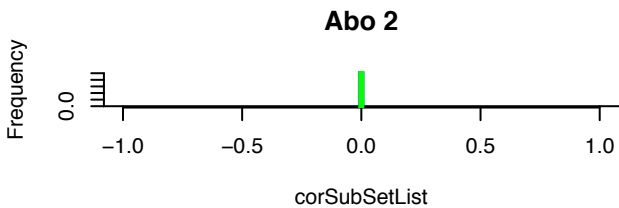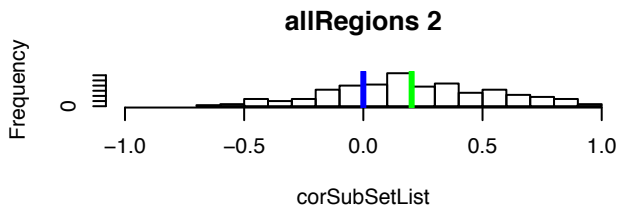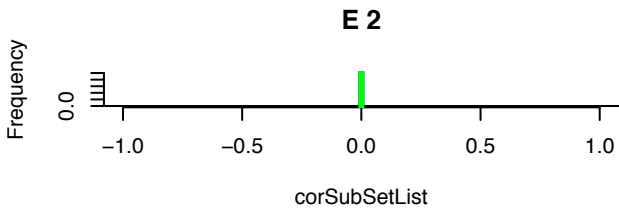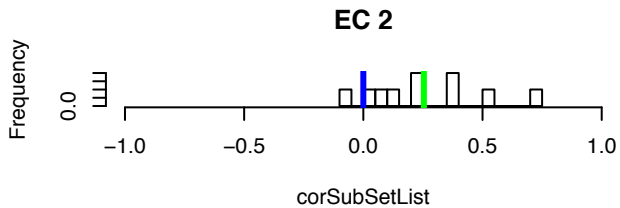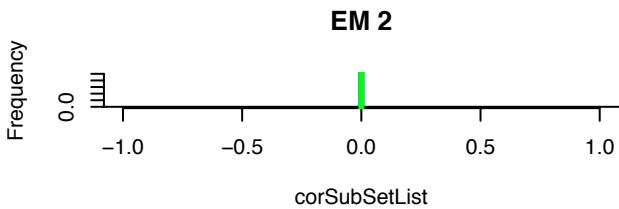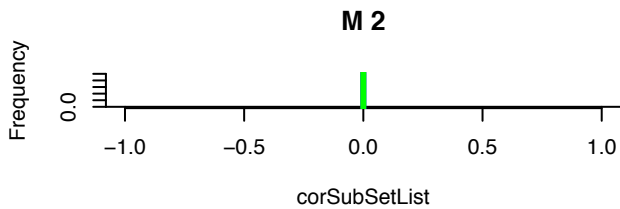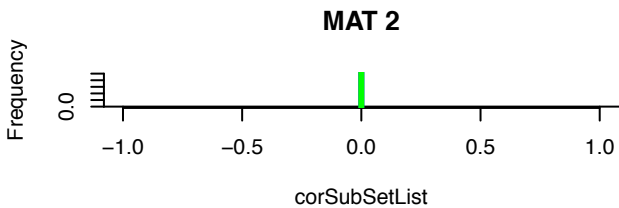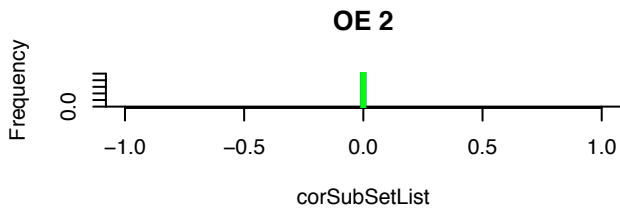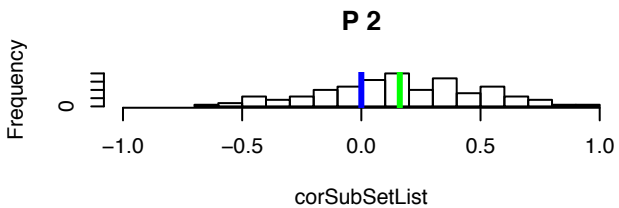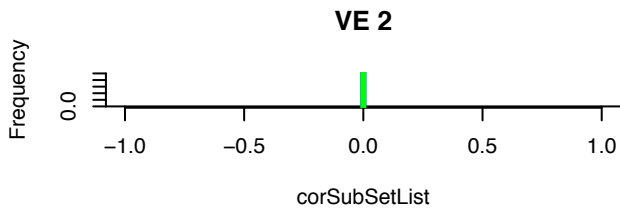

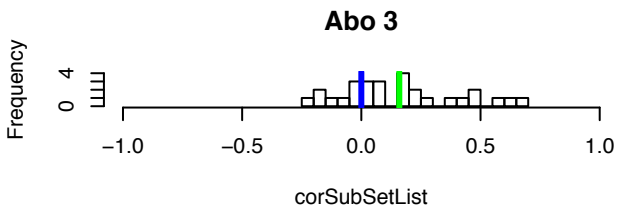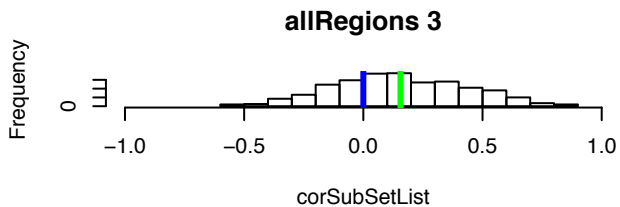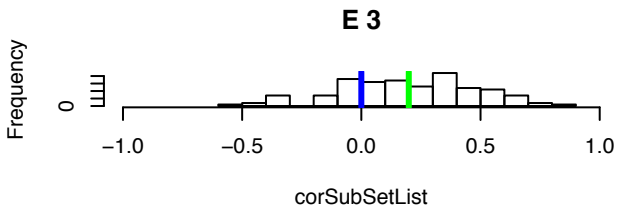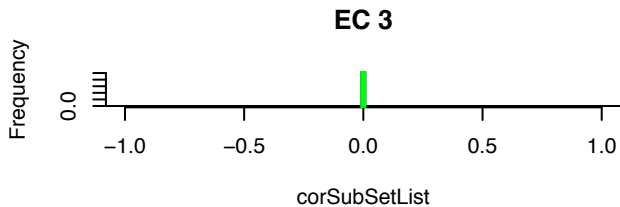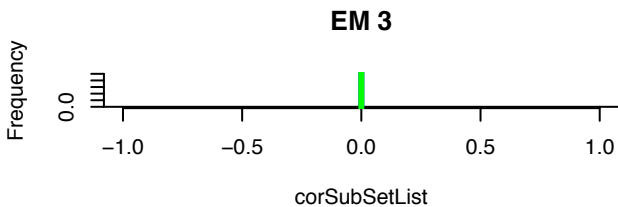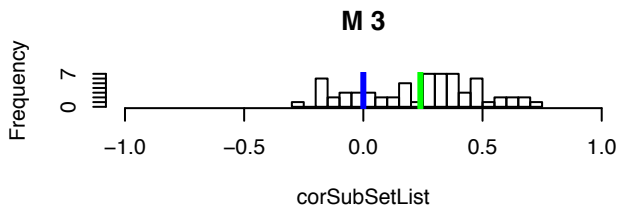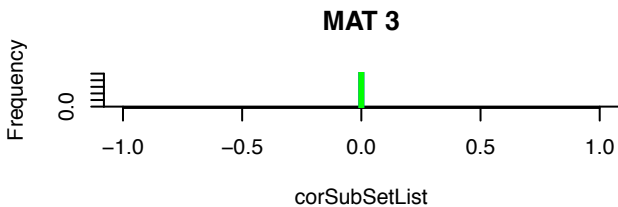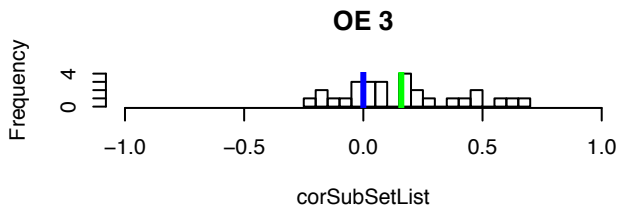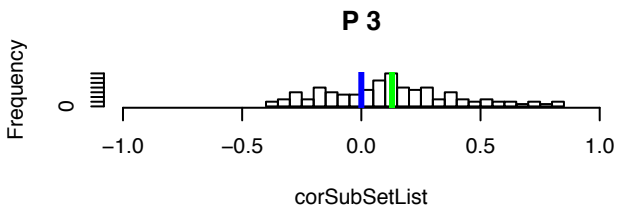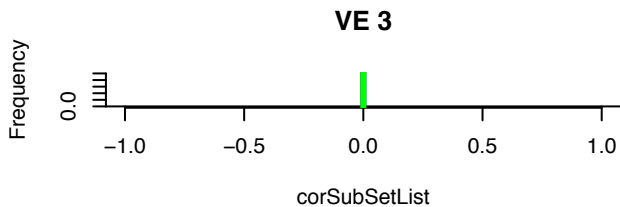

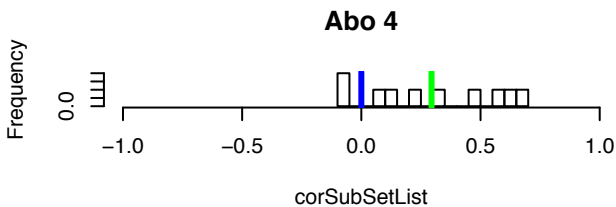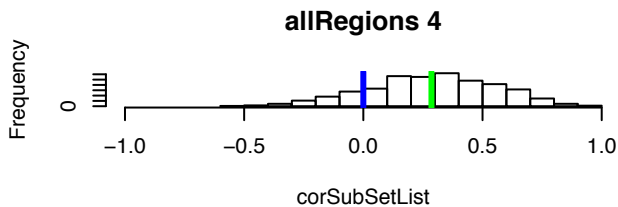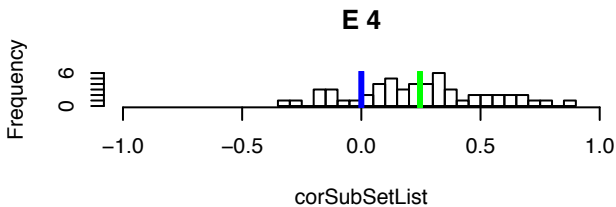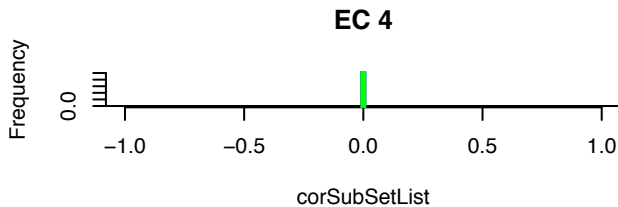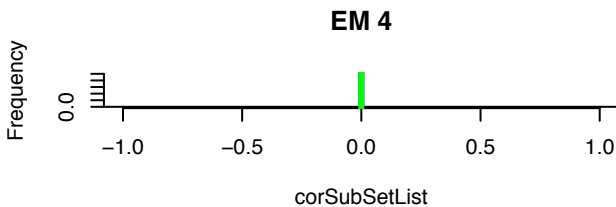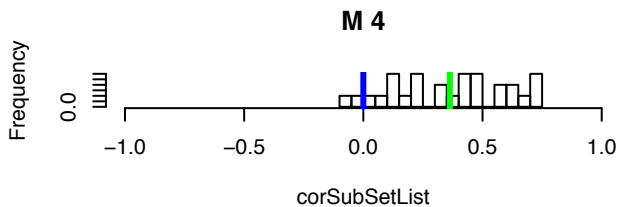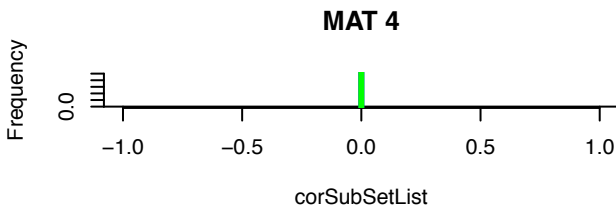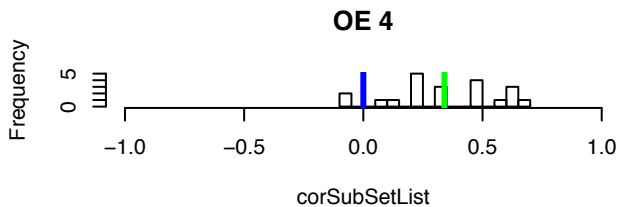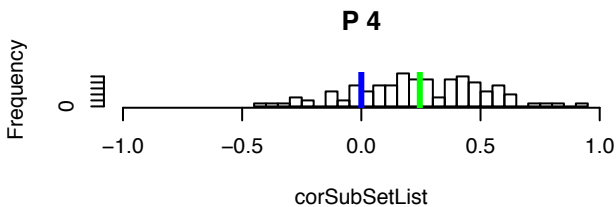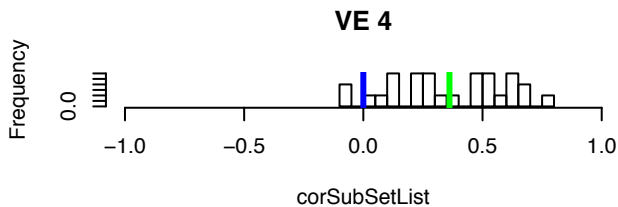

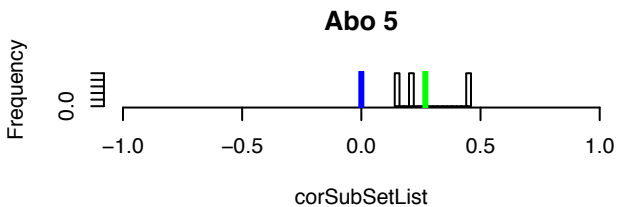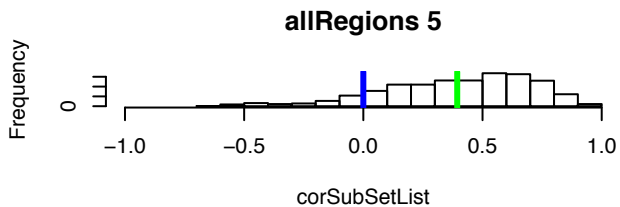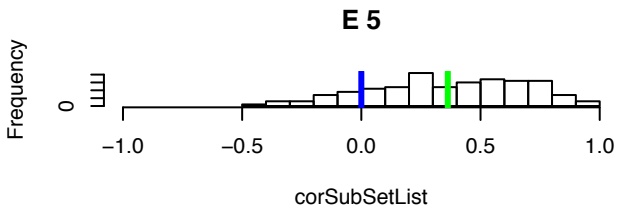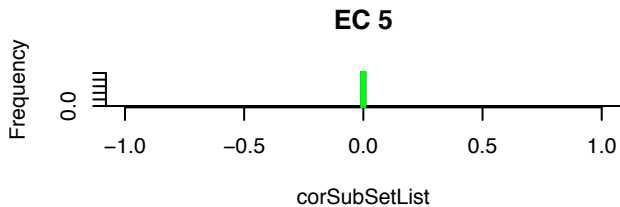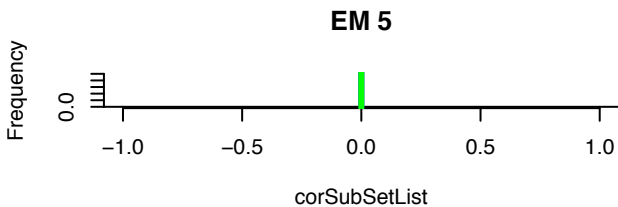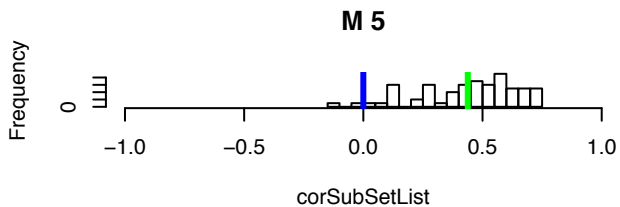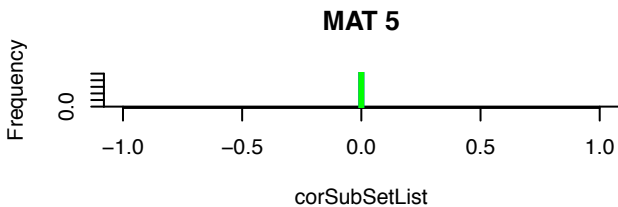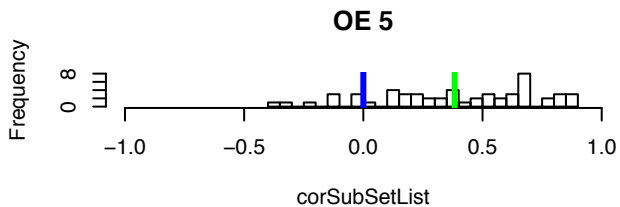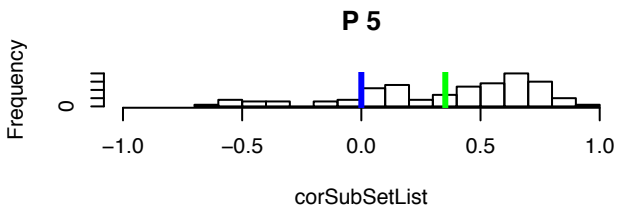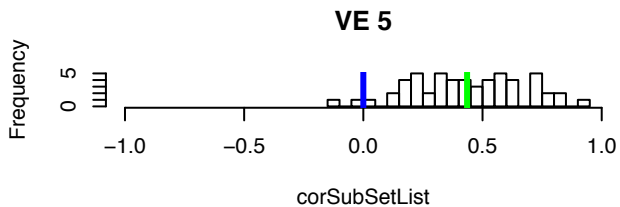

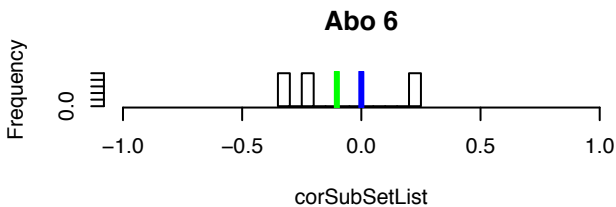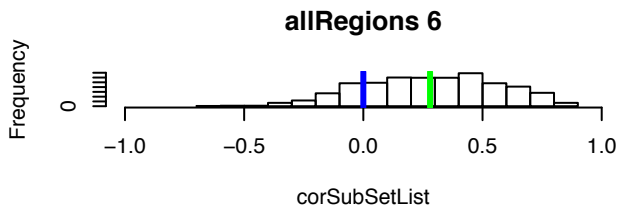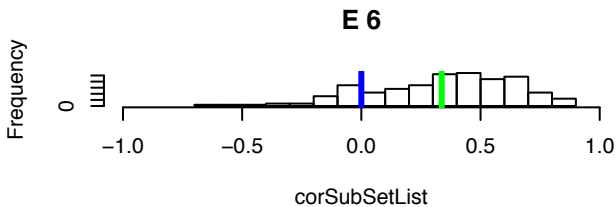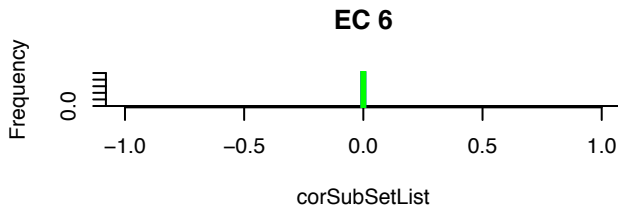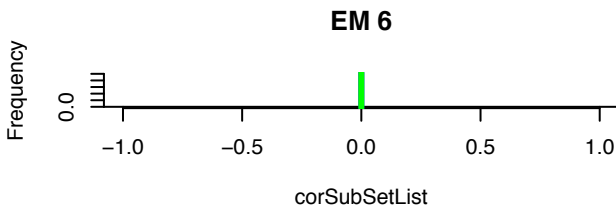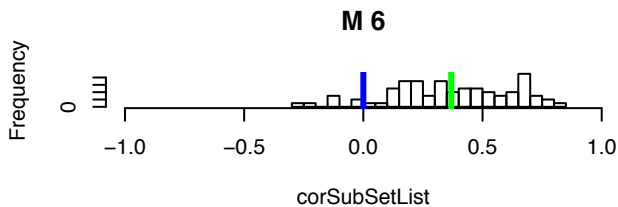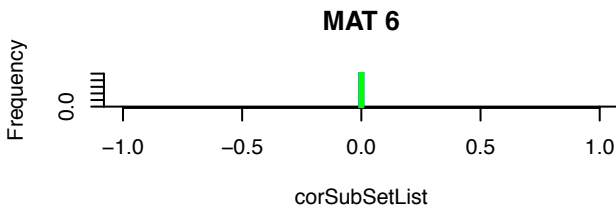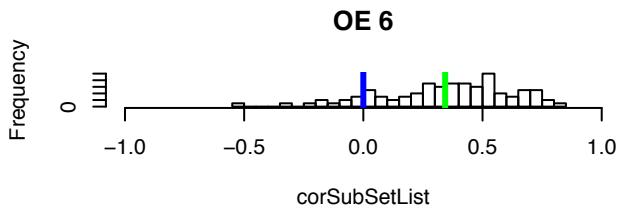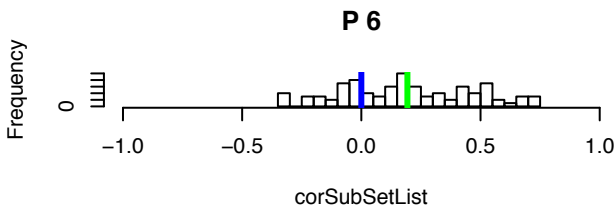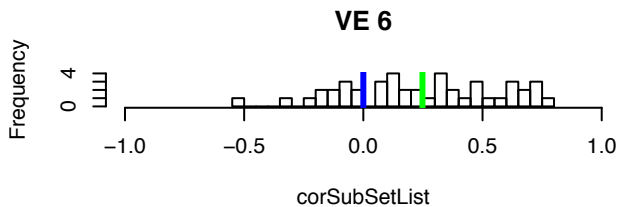

Supplement: Figure S3 — Breeding value correlations by tissue by time. Plotted are histograms of the observed pairwise correlations between breeding values for genes expressed in each of the nine tissues as annotated in the Biotapestry database. Each page represents a different time point (1–6) with the correlations over all annotated genes plotted in the upper right. The blue bar notes 0 and the green bar the average pairwise correlation in that tissue. Following conventions in the Biotapestry database; abbreviations for regions are: Abo, aboral ectoderm; E, endoderm; EC, ectoderm; EM, endomesoderm; M, mesoderm; MAT, maternal; OE, oral ectoderm; P, primary mesenchyme/skeletogenic cell lineage; VE, vegetal. Numbers following these abbreviations refer to time points 1–6 of the present study. (PDF) [file pbio.1001696.s003.pdf]
