# Supplementary material for: The Impact of Gene Expression Variation on the Robustness and Evolvability of a Developmental Gene Regulatory Network
Source: PLoS Biol. 2013 Oct 29;11(10):e1001696. doi: 10.1371/journal.pbio.1001696 (PMC3812118; doi:10.1371/journal.pbio.1001696)
Supplement: Table S2 — Summary of a linear model describing the relationship between variance and time point. The intercept is forced to 0,0. As a result, the estimates are the mean of the total variance at each time point. Importantly, there is no relationship between variance levels and the fraction of sensitive edges described in Figure 4B. (DOC) [file pbio.1001696.s011.doc]

|  | estimate | std. error | t value | Pr (>|t|) |
| --- | --- | --- | --- | --- |
| time1 | 0.6425 | 0.1233 | 5.21 | 0.0000 |
| time2 | 0.4278 | 0.1079 | 3.97 | 0.0001 |
| time3 | 0.1715 | 0.1010 | 1.70 | 0.0902 |
| time4 | 0.3294 | 0.1010 | 3.26 | 0.0012 |
| time5 | 0.2078 | 0.1017 | 2.04 | 0.0416 |
| time6 | 0.2625 | 0.1003 | 2.62 | 0.0092 |
| time7 | 0.2059 | 0.1024 | 2.01 | 0.0450 |
