# Supplementary material for: The Impact of Gene Expression Variation on the Robustness and Evolvability of a Developmental Gene Regulatory Network
Source: PLoS Biol. 2013 Oct 29;11(10):e1001696. doi: 10.1371/journal.pbio.1001696 (PMC3812118; doi:10.1371/journal.pbio.1001696)
Supplement: Table S3 — The relative weightings of each skeletal measure in the first three principle components of skeletal variation. These three axes explain 55.1%, 20.4%, and 13.4% of the total between culture variation in skeletal morphology. (DOC) [file pbio.1001696.s012.doc]

| Measure | PC1 | PC2 | PC3 |
| --- | --- | --- | --- |
| BW | -0.251 | 0.605 | -0.129 |
| ALRT | -0.519 | 0.117 | -0.084 |
| POR | -0.233 | -0.738 | -0.028 |
| PORT | -0.508 | -0.198 | 0.334 |
| ALR | -0.352 | -0.071 | -0.827 |
| BR | -0.481 | 0.177 | 0.424 |
