# Supplementary material for: The Impact of Gene Expression Variation on the Robustness and Evolvability of a Developmental Gene Regulatory Network
Source: PLoS Biol. 2013 Oct 29;11(10):e1001696. doi: 10.1371/journal.pbio.1001696 (PMC3812118; doi:10.1371/journal.pbio.1001696)
Supplement: Table S4 — Male, female, and interaction contributions to between family variation in the principle components of skeletal variation. ** indicates p<0.01 using a standard likelihood ratio test. (DOC) [file pbio.1001696.s013.doc]

| Principle Component | Male Effect |
| --- | --- |
| PC1 | 0.281 |
| PC2 | 0.372 |
| PC3 | 0.421** |
|  |  |
| Principle Component | Female Effect |
| PC1 | 2.951** |
| PC2 | 1.009** |
| PC3 | 1.074** |
|  |  |
| Principle Component | Interaction Effect |
| PC1 | 0.132 |
| PC2 | 0.108 |
| PC3 | 0.057 |
|  |  |
| Principle Component | Residual |
| PC1 | 2.200 |
| PC2 | 0.733 |
| PC3 | 0.385 |
