# Supplementary material for: The Impact of Gene Expression Variation on the Robustness and Evolvability of a Developmental Gene Regulatory Network
Source: PLoS Biol. 2013 Oct 29;11(10):e1001696. doi: 10.1371/journal.pbio.1001696 (PMC3812118; doi:10.1371/journal.pbio.1001696)
Supplement: Table S6 — The weights of each skeletal measure's contribution to the six vectors summarizing skeletal variation produced by the two block partial least-squares analysis. (DOC) [file pbio.1001696.s015.doc]

| Measure | V1 | V2 | V3 | V4 | V5 | V6 |
| --- | --- | --- | --- | --- | --- | --- |
| BW | -0.065 | 0.248 | -0.769 | 0.581 | 0.050 | 0.054 |
| ALRT | -0.496 | 0.366 | 0.438 | 0.321 | 0.569 | -0.030 |
| POR | -0.171 | -0.717 | -0.056 | 0.131 | 0.312 | 0.583 |
| PORT | -0.684 | -0.354 | -0.233 | -0.170 | -0.123 | -0.555 |
| ALR | -0.077 | -0.180 | 0.396 | 0.647 | -0.622 | -0.017 |
| BR | -0.497 | 0.365 | -0.059 | -0.307 | -0.418 | 0.590 |
|  |  |  |  |  |  |  |
