# Supplementary material for: The Impact of Gene Expression Variation on the Robustness and Evolvability of a Developmental Gene Regulatory Network
Source: PLoS Biol. 2013 Oct 29;11(10):e1001696. doi: 10.1371/journal.pbio.1001696 (PMC3812118; doi:10.1371/journal.pbio.1001696)
Supplement: Table S7 — Each of the six pairs of vector found by the two block partial least-squares analysis contributes different amounts to the total correlation between gene expression and skeletal variation. The relative weighting of each of the six pairs of vectors are described by the eigenvalues given in this table. (DOC) [file pbio.1001696.s016.doc]

| V1 | 24898.268 |
| --- | --- |
| V2 | 8526.173 |
| V3 | 7765.634 |
| V4 | 6991.767 |
| V5 | 1505.267 |
| V6 | 797.350 |
