# Supplementary material for: The Impact of Gene Expression Variation on the Robustness and Evolvability of a Developmental Gene Regulatory Network
Source: PLoS Biol. 2013 Oct 29;11(10):e1001696. doi: 10.1371/journal.pbio.1001696 (PMC3812118; doi:10.1371/journal.pbio.1001696)
Supplement: Table S8 — The top 10% of gene expression contributions to the first 2B-PLS axis. The column “Gene_Cluster_Time” gives the gene name followed by the cluster number (see Text S1 – “Gene expression DASL processing”) followed by the time point at which the expression measurements were taken. (DOC) [file pbio.1001696.s017.doc]

| Gene_Cluster_Time | Weight |
| --- | --- |
| FoxO_1_5 | 0.241 |
| HesC_1_1 | 0.236 |
| Tel_1_1 | 0.218 |
| soxb1_1_1 | 0.193 |
| SoxC_1_1 | 0.191 |
| SM30.E_1_5 | -0.187 |
| SM30.E_1_6 | -0.185 |
| B.catenin_1_1 | 0.168 |
| otx_1_1 | 0.166 |
| otx_1_5 | 0.162 |
| Fmo2_1_6 | -0.157 |
| SM50_1_3 | -0.155 |
| Pmar1_1_3 | -0.151 |
| tbr_1_1 | 0.134 |
| Chordin_1_2 | 0.127 |
| Wnt8_1_1 | 0.126 |
| Blimp1.Krox_1_5 | -0.118 |
| nodal_1_1 | 0.117 |
| Gsk.3_1_1 | 0.114 |
| Fmo2_1_5 | -0.112 |
| Lefty_1_1 | 0.111 |
| SM27_1_3 | -0.105 |
