# Supplementary material for: The Impact of Gene Expression Variation on the Robustness and Evolvability of a Developmental Gene Regulatory Network
Source: PLoS Biol. 2013 Oct 29;11(10):e1001696. doi: 10.1371/journal.pbio.1001696 (PMC3812118; doi:10.1371/journal.pbio.1001696)
Supplement: Table S9 — By combining the gene expression contributions to each of the six axes weighted by the eigenvalue corresponding to each axis, we get a measure of the overall contribution of each gene_time expression to the overall relationship between gene expression and skeletal variation. Above are the top 5% of total the weighted contributions of expression measurements to the overall correlation between gene expression and skeletal variation. (DOC) [file pbio.1001696.s018.doc]

| Gene_Cluster_Time | Total Weight |
| --- | --- |
| Lefty_1_1 | 8647.936 |
| FoxO_1_5 | 8385.755 |
| Tel_1_1 | 7954.273 |
| HesC_1_1 | 7573.943 |
| SoxC_1_1 | 6953.516 |
| Pmar1_1_2 | 6659.644 |
| Chordin_1_2 | 6353.916 |
| SM30.E_1_5 | 6336.727 |
| SM30.E_1_6 | 6303.137 |
| Pmar1_1_3 | 6230.714 |
| soxb1_1_1 | 6221.594 |
