# Supplementary material for: The Impact of Gene Expression Variation on the Robustness and Evolvability of a Developmental Gene Regulatory Network
Source: PLoS Biol. 2013 Oct 29;11(10):e1001696. doi: 10.1371/journal.pbio.1001696 (PMC3812118; doi:10.1371/journal.pbio.1001696)
Supplement: Text S1 — Supplemental methods. (PDF) [file pbio.1001696.s020.pdf]

Supplemental Methods for “The impact of gene expression variation on the robustness and evolvability of a developmental gene regulatory network”

David A. Garfield, Daniel E. Runcie, Courtney C. Babbitt, Ralph Haygood, William J. Nielsen, and Gregory A. Wray

CONTENTS

Animal rearing and sample collection 1
Measurements of larval morphology 2
Analyses of larval morphology 2
Estimates of additive genetic variances and covariances 3
Bayesian estimates of quantitative genetics parameters 6
Additive genetic variances over development 10
Genetic covariances over development 11
Pre-processing raw bead-level data from DASL 12
Expression validation 19
Supplemental References 22

EXTENDED EXPERIMENTAL PROCEDURES

Animal rearing and sample collection

Adult sea urchins were collected during a single dive from a population in the Santa Barbara channel, shipped overnight to Duke, and held at 12°C in artificial sea water (Coralife, Oceanic Systems Inc.) for <48 hr before spawning. Gametes were obtained and fertilization carried out following standard procedures [1]. All cultures were fertilized simultaneously and checked to ensure >95% fertilization by the criterion of raised fertilization envelopes. Samples were taken only after hatching for families in which <95% fertilization occurred to ensure that measurements were not confounded by the presence of maternal mRNAs in unfertilized eggs. Zygotes (36 families for TP3-7 and 25 for TP1,2) were split into replicate cultures in a

randomized design and raised in artificial seawater at 12°C. At seven time points (10, 18, 24, 28, 38, 45, and 90 hr post-fertilization), samples were collected from each culture for gene expression analysis, concentrated by gentle centrifugation, the pellet transferred into 600μL of buffer RLT (Qiagen, Valencia, CA), homogenized, and stored at -80°C. Samples for morphology were collected from the final time point (90 hrs), fixed for ~30 min in a 4% PFA/artificial sea water solution, transferred to ice-cold MeOH, and stored at -20°C.

Measurements of larval morphology

To quantify morphological variation, we adapted standard measures [2]. We mounted each larva in PBS solution, collected a z-stack of 3 μm optical sections through the entire specimen on a Zeiss Axioskop 2 with Axiovision v4.6, and exported the image to ImageJ v1.4 (Rasband 1997-2009). We then recorded the 3D-locations of 8 landmarks in the skeleton of each larva (Figure S4) and converted these into 9 length measures using custom Python (www.python.org v2.1) scripts: 6 rod lengths [left and right body rods (BR), left and right post-oral rods (POR) and left and right anterolateral rods (ALR)], as well as 3 summary "size" measures [body width at the triradiate junctions (BW) and left and right total length from tip of the post-oral rod to tip of the body rod (PORT)]. We found no evidence for developmental asymmetries, so we averaged the measures from the right and left side of each larva. Because gene expression measures were taken at the level of cultures and not individuals, we averaged the measures from all larvae in a single culture.

Analyses of larval morphology

We took two approaches to evaluating the relationship between gene expression and larval morphology. In the first analysis, we sought correlations between the expression of specific genes at specific stages (gene-times) and principle components of skeletal variation (See Table S5 for the full set of correlations). The first three principle components (Table S3) together explain 89% of the total variation, with the first Component explaining the majority of the variation (55.1%). These PCs show significant parent-of-origin effects based on comparisons

between nested models with and without the factor of interest (Table S4). Dam effects are particularly pronounced as one might expect given the documented relationship between variation in maternal egg quality and skeletal shape in echinoderms [3,4]. For each gene and development time point sampled, we calculated the Pearson correlation coefficient with each principle component of variation in the larval skeleton. At a false discovery rate of 5%, three such correlations were significant: *SM30E* at time point 4 is positively correlated with overall length (PC1), while *FoxB* at time point 4 and *Hex* at time point 3 correlate with the ratio of arm length to body length (PC3). In addition to the genes just mentioned with q-values <0.05 for a single time point, six genes are overrepresented among the 234 gene-time point combinations with nominal p-values <0.05. Although each time point is individually less strongly correlated with skeletal variation, these correlations extend across multiple time points, making the expression profile as a whole significant by permutation (p <0.05). These genes are: *Msp130*, *SM30E*, *SM50*, *C-lectin*, *Dkk*, and *Su(H)*. In our second approach, we examined the relationship between the set of gene expression measures as a whole and skeletal variation using a Two Block Partial Least-Squares analysis (2B-PLS) [5,6].Formally, the analysis is a singular value decomposition of the portion of a variance-covariance matrix in which all of the rows represent the elements of one dataset and all of the columns represent elements from the other. The level of statistical significance of the overall RV-statistic is obtained by permutation as detailed in the source papers. Information concerning the genes that contribute the most to the correlation between skeletal variation and gene expression variation can be found in Tables S6-S9. The full set of 2B-PLS weights are available upon request.

Estimates of additive genetic variances and covariances

The proportion of the total phenotypic variation within a population that can be explained, in a statistical sense, by genetic background-independent contributions of genetic variation is the *additive genetic variance* of a trait, and it is the size of this variance relative to overall phenotypic variance that puts bounds on the efficacy by which selection can change the mean

value of a trait [7]. We estimated additive genetic variances and covariances between traits based on measures of parental effects from a NCII cross with pooled samples. Following the standard partition of phenotypic variation (σ_P) [7]:

$$\sigma^2_P = \sigma^2_{A+} \sigma^2_{I+} \sigma^2_E \qquad (1)$$

where σ^2_A is the additive genetic variance, σ^2_I is the non-additive genetic variance and σ^2_E is the environmental variance, including any interaction between genetics and environment, σ^2_A is proportional to the narrow-sense heritability (h^2):

$$h^2 = \sigma^2_{A\,/}\,\sigma^2_P \qquad (2)$$

and thus also proportional to the expected response to selection ($\Delta\mu$) across generations:

$$\Delta\mu = h^2 S = \sigma^2_{A\,/}\,\sigma^2_P\ S. \ (3)$$

Our mating scheme of male and female parents followed the North Carolina II (NCII) breeding design [7,8]. When parents are fully outbred and randomly sampled from a wild population, this cross-classified design can be used to efficiently estimate the population's additive genetic variance, as well as other useful quantitative genetic parameters such as maternal effects and, in some situations, the dominance variance. The NCII design is useful when both parents can be mated to multiple parents of the opposite sex. This is straightforward in species with external fertilization, like sea urchins, because gametes from each parent can be collected and divided into multiple batches before fertilization, thus eliminating any effects from the order of matings. In an NCII cross, offspring that share the same female parent but have different male parents are maternal half-sibs, while offspring that share the same male, but not female parent, are paternal half-sibs. Thus, the variance in male (or female) effects in a linear model relating offspring phenotype to parent identity is an estimate of the population covariance of paternal (PHS) (or maternal (MHS)) half sibs (chapter 20 of ref [7]). Assuming epistatic variance is

negligible, half-sib covariances can be related to additive genetic variances in the following ways:

$\sigma^2_m = cov(PHS) \simeq \sigma^2_A/4$

$\sigma^2_f = cov(PHS) \simeq \sigma^2_A/4 + \sigma^2_{Mat}$ (4)

where σ^2_m is the male variance, σ^2_f is the female variance and σ^2_{Mat} is the variance in maternal effects, coming from either maternal-genetic or maternal-environment factors. (An interesting question arises if epistatic variance is not negligible, as may be the case within a GRN. In a more full formulation $\sigma^2_m = cov(PHS) \simeq \sigma^2_A/4 + \sigma^2_{AA}/16$ where σ^2_{AA} is the additive-by-additive epistatic variance. Though the epistasis variance in this formulation is a relatively minor contributor to the covariance among half sibs, it could influence the extent to which observed genetic variation can be directly acted upon by selection. In any event, σ^2_{AA} still reflects real genetic variation segregating in the population.) Thus, both male and female variances are estimates of the total additive genetic variance. They are not, however, identical to it, and, if the female variance is considerably higher than the male variance, the difference can be used as an estimate of the influence of maternal effects. Similarly, individuals that share both the same male and female parent are full sibs. The covariance of full sibs, minus the sum of the covariances of paternal and maternal half-sibs, is estimated in the linear model by the male x female interaction variance (σ^2_I), which serves as an estimate of 1/4 the dominance variance (σ^2_D):

$\sigma^2_I = cov(FS) - (cov(PHS) - cov(MHS)) \simeq \sigma^2_D/4$ (5)

Unfortunately, the size of the cross rather limits the accuracy with which interaction effects can be estimated. Since we raised embryos from each male-by-female cross in two separate cultures, the derivation of male and female variances in our experiment more closely follows that in Comstock and Robinson, where "*k* members of each progeny [offspring from a particular

cross] are grown in each of *r* plots [culture dishes]". From the Analysis of Variance shown in Table 2 in this paper, the male variance (σ^2_m) can be calculated as the difference in expected mean squares of the the Male and Males x Females factors:

$$EMS(Males) = \sigma^2 + k\sigma^2_p + rk\sigma^2_{fm} + rkn\sigma^2_m$$

$$EMS(Males\ x\ Females) = \sigma^2 + k\sigma^2_p + rk\sigma^2_{fm}$$

$$\sigma^2_m = (EMS(Males) - EMS(Males\ x\ Females)) / rkn \quad (6)$$

where *n* is the number of males. The derivation of female variance (σ^2_f) is similar. Our analysis differed from the ANOVA derivation in Comstock and Robinson in that we did not divide the parents into multiple sets (*s* = 1) and that we did not measure gene expression on individual larvae, but on pooled larvae from the same culture. Thus, we could not estimate an individual variance (σ^2 in Table 2 of reference [8]). Since the individual variance contributes equally to the EMS of Male, Female and Male x Female effects, the fact that we could not estimate this parameter did not affect our estimate of male or female variances (equation 4). However, by pooling larvae to collect RNA, we could not measure variation among individuals within a culture, and thus could not calculate the total phenotypic variation (σ^2_P) or "heritability" in gene expression. Importantly, however, by pooling thousands of larvae instead of measuring just a few individuals, we improved our estimates of additive and non-additive genetic variances, by reducing sampling error on culture means.

Bayesian estimates of quantitative genetics parameters

Model specification

While the Analysis of Variance presented by Comstock and Robinson (Table 2 in reference [8]) is sufficient to estimate genetic variances in our experiment, ANOVA methods are not well suited for estimating error terms or significance in the face of missing data. We therefore converted their standard mixed-effect linear model into a Bayesian hierarchical mixed-effect model by

adding priors on the genetic and residual variances and fitting the model using a Gibbs sampler, implemented in the *MCMCglmm* package in *R* [9]. We took this approach for two reasons. First, due to filtering for quality control of the gene expression measures, certain samples with low quality expression measurements were removed, resulting in an unbalanced design. Likelihood-based methods, such as REML and Gibbs samplers inherently tolerate unbalanced designs, while ANOVA methods require complicated adjustments [7]. Second, since our sample sizes were small for estimating variances, asymptotic closed form confidence-interval estimates on variance components such as those produced from REML or ANOVA methods, are not reasonable, and return confidence intervals that span zero for low variance estimates. Bayesian credible intervals can be much more interpretable in these situations as they do not depend on asymptotic assumptions and can be enforced to be positive and asymmetric. We estimated genetic variances with the following linear hierarchical mixed effect model:

$$
\begin{aligned}
&y_{i,j,k} = u + m_i + f_i + I_{i,j} + e \\
&u \sim N(0,10^6) \\
&m_i \sim N(0,\sigma_m^2) \\
&f_i \sim N(0,\sigma_f^2) \\
&I_{i,j} \sim N(0,\sigma_{mf}^2) \\
&e_{i,j,k} \sim N(0,\sigma_e^2) \\
&\sigma_m^2 \sim InvGamma(2,0.001) \\
&\sigma_f^2 \sim InvGamma(2,0.001) \\
&\sigma_{mf}^2 \sim InvGamma(2,0.001) \\
&\sigma_e^2 \sim InvGamma(2,0.001)
\end{aligned}
$$

where y_{ijk} is the phenotype of the kth culture of the cross between male i and female j, u is the population phenotypic mean, m_i and f_j are the additive effects of male i and female j, I_{ij} is the non-additive effect of the particular genetic combination of male i with female j, and e_{ijk} is the residual deviation of the pooled sample from culture k of male i and female j. The terms m_i, f_j, I_{ij} and e_{ijk} are all modeled as random effects, drawn from normal distributions with mean zero and

unknown variance. These unknown variances are given inverse-gamma priors.

Prior choice

The inverse gamma prior is a common choice for variance components in linear models as it is conjugate to the normal likelihood and is the default prior in *MCMCglmm* [9,10]. For the results presented in the main article, we used a quasi-empirical Bayes approach and chosen as a prior a diffuse inverse gamma centered at 10% of the variance in the expression of each gene. This prior distribution states that our prior belief is that each variance-component is non-zero, but is small and right-skewed with a relatively thick tail. The strength of prior is uniform across all genes, reflecting our lack of prior knowledge about differences in genetic variance components among genes. This facilitates comparisons of posterior distributions among genes as the strength of the prior should be identical in all cases. Our prior was selected for two reasons: it has a broad distribution reflecting our relative lack of prior information on the genetic influences on the expression of each gene, and a previous study [11] found that the mean broad-sense heritability of gene expression to be ~0.5, suggesting individual male and female variances should be in the range of 12.5% [1/4 total additive genetic variance, equation (4)]. Nonetheless, we examined the influence of our chosen prior on the results extensively. For genes with moderate levels of expression variation, the results are largely insensitive to both quantitative changes in size and shape of the inverse-gamma prior as well as to changes in the form of the prior; our results are highly correlated both with Bayesian estimates obtained using a folded central t-distribution [10], and to estimates obtained with REML models fit using both the R package lmer [12] and ASReml [13]. Results from all three methods were highly similar.

Model fitting

We fit the above model to the normalized expression of each gene at each of the 7 time points individually using the *MCMCglmm* function in the *MCMCglmm* package v. 2.02 in R. After a burn-in period of 5,000 samples, we collected 10,000 posterior samples of all parameters and

variance components, thinning the chain every 10 samples. Model convergence was checked by assaying the autocorrelation among each variance parameter separately. Posterior distributions of each variance parameter were summarized as the mean and 5% and 95% quantiles of the posterior samples. In most analyses (those not noted otherwise), the expression of each gene at each time was first scaled by the mean expression of that gene at that time point. This was done so that the square root of the variances fit by the quantitative genetics models could be read in the same scale as the mean.

Model significance

We took two approaches for assessing the statistical significance of male, female, and interaction contributions to gene expression variation for each gene at each time point. First, we used permutation tests. To assess the significance of male effects, we fit the full model using ASReml and compared the estimated size of the male effect to 1000 permuted datasets in which the male labels were randomly assigned to each culture. The statistical significance of female effects, but not interaction effects, were tested similarly. Second, we used the output of *MCMCglmm*. To test the importance of the male effects, we fit refit a reduced model by dropping the male (m_i) and male x female (I_{ij}) effects. We compared the full model (with male effects) and the reduced model (without male effects) using the DIC calculated by the *MCMCglmm* function. Genes for which the DIC increased by more than 7 were deemed to have a significant male effect. Female and interaction effects were tested similarly. As the results of both methods were highly similar, we made use of the second method in the analyses reported in the main text. To test for non-maternal relationships between the expression of candidate genes and skeletal variation, we contrasted two models using the R package lme4. In the first model, we fit each of the first three principle components of skeletal variation as a function of female parent (shape ~ Female). In the second model, we fit shape as a function of both female parent and an upstream gene (shape ~ gene + Female) and contrasted the two models using a standard likelihood ratio test. In most cases we did not see significant support (or, often, any support) for models that included more than just female parent. The exceptions were as follows: for *SM30E*,

we found significant contrasts ($p < 0.01$) for PC1 and PC3 at time point 5 and for PC1 at time point 4; for *FoxO*, we found a significant contrast for PC1 at time point 5; and for both *C-lectin* and *Msp*130, we found a significant contrast for time point 5 for PC1 and PC3.

Additive genetic variances over development

The sum of male and female variances provides an estimate of one half of the additive genetic variance (see above). Unless otherwise noted, data were first scaled by the mean. Therefore, the square root of the additive genetic variance can be interpreted similarly to be a coefficient of variation measure. Using maternal variances as a contribution is a concern because this may inflate additive genetic variances, especially at early time points. However, we justify our decision on several grounds. First, because zygotic expression is very low at 10 hr and, to a lesser extent, 18 hr, relying only on paternal contributions will vastly underestimate levels of genetic variation; clearly some portion of the differences between pools of embryos/larvae has a genetic component. Second, previous studies have demonstrated that there are genetically based differences in maternal contributions to the eggs even between very closely related species. Third, to the extent possible we controlled for environmental differences between females by using females of roughly the same age taken from the same location. Using 2*(male effect + female effect) to estimate additive genetic variance (Figure S1A), we see a clearly heterogenous distribution over development (Kruskal-Wallis, $p = 2.9 \times 10^{-5}$, $\div^{2} = 61.21$, df = 6) with early development (the first two time points) showing significantly higher levels of additive genetic variance than later development (var = 0.572 *vs* 0.132 $p = 1.94 \times 10^{-7}$ Wilcoxon). Importantly, we obtain qualitatively similar results when we examine only statistically significant male effects (Kruskal-Wallis test $p = 0.0015$, var = 0.038 *vs* 0.026 $p = 0.09$ Wilcoxon) or when we look at mean parental contributions for genes only during the times in which they are known from prior research (see above) to be involved in regulatory interactions (Kruskal-Wallis test $p = 0.039$, 0.18 *vs* 0.07 $p = 0.051$ Wilcoxon).

Genetic covariances over development

Owing to the relatively small size of our cross, we cannot effectively estimate the genetic contributions to covariances (especially not for individual genes). However, using our best estimates, we may be able to say something about the relative magnitudes of the covariance matrices as well as how structured they are. These analyses suggest that genetic covariances are structured quite differently at time point 1 than at later stages. The results should, needless to say, still be interpreted with caution.

Using a two-gene extension of the basic model, we are able to estimate the genetic covariances for each pair of genes at each time point and from this to construct, piece-meal, a standard genetic covariance matrix (G-matrix). At no stages of development are genetic covariances between interacting genes significantly different than for random pairs of genes, which likely demonstrates the limits of our power to estimate covariances. Interestingly, however, the mean strength of the genetic covariances between genes when they are interacting changes noticeably over development, with significantly greater covariances in the first two stages of development than in subsequent stages (cov = 0.016 vs. 0.00010, $p < e\text{-}15$ t-test). Importantly, however, the differences among time points are no longer statistically distinguishable when the first time point is removed from consideration (with Time Point 1 $p = 3.44e\text{-}10$; without Time Point 1 $p = 0.06$ Kruskal-Wallis Test) highlighting the extent to which time point 1 is structured differently than are later stages of development. Analyses of genetic correlation matrices give similar results with genetic correlations in the first stage being statistically distinct (and higher) than in subsequent stages (data not shown).

As a metric of constraint, we can also examine the variance of relative magnitudes of eigenvalues for the G-matrix at each sampled time in development. High variances indicate that the G-matrix is constrained to evolve primarily in one or a few directions, while a low variance (all eigenvalues having similar magnitudes) is indicative of a relative lack of constraint.

Comparing the variances in eigenvalues is complicated in our case by the fact that there are different numbers of genes and parents both detected and active at different stages of development. As a result, the G-matrices have different dimensionalities at different stages of

development. To circumvent this problem, we sub-sampled each G-matrix 100 times to generate sub-matrices with dimensionality equal to the minimum number of eigenvalues of any G-matrix in the analysis. We then took the average of the resulting variances. Quantitatively similar results are obtained if one samples instead the eigenvectors corresponding to the n largest eigenvalues, where n is the dimensionality of the smallest G-matrix.

To assess the statistical significance that the calculated variances were greater than expected from random matrices, we recreated the G-matrices directly from the breeding values and compared the variance of this matrix to 1000 G-matrices resulting from permutations of the breeding values. We used the 95% intervals to evaluate the claim that the variances in eigenvalues are statistically different from one another at different times in development. We used 1000 subsamples of each G-matrix to calculate these intervals.

At all stages, for both expressed and active genes, the complexity of the G-matrix is significantly greater than expected by chance (permutation test, $p < 0.01$). However, the variance in eigenvalues is not equal at all stages. When the G-matrices are restricted to only active genes, a clear pattern emerges with the G-matrices being more restrictive in the first sampled stages than in later development [(Var(eigenvalues) = 0.083, 0.080, 0.024, 0.049, 0.023, 0.019] though only the first stage is distinct by permutation. Interestingly, the complexity of the G-matrices are both lower and more even across development when we consider only those genes not known to participate in molecular interactions at that time [Var(eigenvalues) = 0.039, 0.045, 0.016, 0.05, 0.038, 0.034, 0.011], indicating decreased constraints on the evolution of gene expression patterns outside of times in which a gene product is required for specific molecular interactions.

Pre-processing raw bead-level data from DASL

DASL differs from other, more common, microarray platforms in a number of ways that necessitate the use of different background correction, summarization and normalization methods [14]. For example, DASL uses ~30 individual beads per sample for each probe set,

rather that the typical 1-3 spots on more standard microarrays, and rather than using a mismatch probe-set, as is used in Affymetrix arrays, background normalization relies on a set of 27 bead-types with no complement in the target genome. As with all platforms for measuring gene expression, quality control and normalization steps must be taken before the data can be used in subsequent analyses. Our principle concern was to remove artifactual biases that might induce correlations among measures of different genes, or among measures of the same gene in different genetic backgrounds, as these biases would affect our downstream genetic analyses. To ensure a high standard of quality in the data, we wrote a customized pipeline in R (R Development Core Team 2009) for processing the raw data using many of the classes and methods from the *beadarray* package of *Bioconductor* [15,16]. Our pipeline included the five steps outlined below. In addition, we performed quality control throughout to: flag and remove suspicious samples and probes; identify cases of potential sequence polymorphisms under probes based on probe intensities in gDNA samples; identify sets of probes that do not appear to measure the same transcript; and choose an appropriate gene for normalization.

Step 1: Mask problem areas in each array

Array-based formats can suffer from spatial artifacts - regions of the assay surface that produce consistently different intensity readings due to camera or laser shadows, loading error, or inherent biases around the edge of the array. If not accounted for, these effects can badly skew resulting analyses, even in cases like the DASL assay where each gene expression level is interrogated by a large number of probes [17]. We used the adaptation of Harshlight to Illumina BeadArrays, *BASH*, implemented in the Bioconductor package *beadarray* [17] with the following modified parameters: bgcorr=”median” as recommended for SAM arrays, diffsig = 0.001, and no imputation within outlier regions. The BASH algorithm relies on the variance in expression measures within each bead-type as the statistic to identify spatial effects. We used log2-transformed values to identify outlier beads, as recommended [17]. We found that log2-transformed intensities better identify outliers at the lower end of the distribution than does

Ilumina's recommended procedure (data not shown). We also chose to use an outlier cutoff of 2 mean absolute deviations (MADs) from the bead median for each bead-type in each sample. This is more conservative than the *beadarray* default and Illumina recommendation of 3 MADs, but we find that this tends to eliminate more spurious beads. Figure S5 shows several examples of the spatial distribution of residual intensities in the red and green channels. Note that some, but not all, spatial effects are consistent between the two channels and that not all arrays have significant spatial effect issues. Although some aspects of masks appear to be shared by most wells of the same plate (batch of 96 samples), most spatial artifacts appear to be individual well-specific. Thus we chose not to apply the same mask to all wells of a plate. We also observed that certain rows and columns within a plate suffered more from imaging artifacts than other wells. Overall, 8.7% (957,562 of 11,906,519) of beads were removed by the masks. Of the remaining beads, 20.7% (2,470,602 of 10,948,957) were removed based on the 2 MAD from the median bead-specific cutoff. The standard Illumina algorithm of identifying outliers that lie more than 3 MADs from the median would have removed 14.7% (1,755,714 of 10,948,957) of the remaining beads. Figure S6 shows the distribution of number of remaining beads per bead-type per sample. No probes ended up with less than 5 beads in any sample, and thus no probes were removed based on too few measures within a sample.

Step 2: Average two colors

The DASL assay uses two probes to target each sequence, one labeled with Cy3 (green) and one with Cy5 (red). In principle, as the oligo probes conjugated to each dye are identical, the red and green channels should show identical intensities. However, as with other array-based platforms (*e.g.*, [18]), the dyes perform differently across the intensity spectrum (Figure S7).

There is no clear precedent in the literature as to how to deal with the two channels in a DASL data set. Illumina recommends adding the two channels together (also see [16]), while (Dunning, personal communication) recommend using only the green channel. In microarrays, and when using Illumina's similar GoldenGate technology for allele-specific expression, a local regression-based correction of the two channels is frequently applied to the data to account for

this intensity-dependent bias, and this was the path we followed. We compared the performance of the red channel alone (R), the green channel alone (G), the mean of the red and green channels (R + G), as well as two loess-transformed [19] averages: the mean of the red and the transformed green channels (R + Gc) and the mean of the green and the transformed red channel (G + Rc). We calculated a local regression of one channel onto the other, using the R function *loess*, with a bandwidth of 0.40 [19], then used this empirical function to transform the first channel so that its intensity-dependent distribution was comparable to that of the second channel. The R+Gc channel consistently performed as well or better than the other channels in terms of dynamic range, the correlation of intensities across technical replicates, and the correlation of intensities among different probes targeting the same transcript. Therefore, we used the R+Gc summary statistic as our measurement of bead intensity for all analyses.

Step 3: Correct background

Background correction involves correcting for non-specific hybridization and for differences in the camera intensity among arrays. Differences in background levels among samples, if left uncorrected, lead to strong positive correlations between low-expressed genes. We used the following modified version of Illumina's background-subtraction method to identify unreliable probes with low intensities and remove correlations due to variation in background noise among samples. The DASL assay includes 27 non-specific bead-types intended to measure the background intensity of an array. We used the negative control beads to determine the background level for each sample. Under close inspection, we observed that the 27 non-specific background probes did not all estimate the same "mean background" intensity: some consistently recorded a much higher intensity than the others. However, the relationships between the intensities of the various background probes in different samples were fairly linear. Therefore, the mean of the background probes was a poor estimate of the true background intensity of each sample. Instead, we calculated the slope and intercept between the background probes in all pairs of samples (using the function *lm* in *R*) and adjusted the intensities of all probes so that the background probes overlapped (Figure S8). We then re-

calculated the distribution of negative control intensities, and chose a value greater than 90% of the negative control intensities to be our “zero” value. All signal values less that this value were set to 1, and this constant was subtracted from the remaining intensity values in each sample. We experimented with other model-based background correction methods specifically adapted for Illumina BeadArray data including an implementation of the *normexp* method from *RMA* [20,21,22], an extension of the *normexp* method to include information from the negative control beads (MBMB) [23], and an adaptation of the *vsn* method (*VST*) [24]. However, we found that these methods required more data to generate an accurate fit to a background-signal model than the 428 probes in our assay could provide and thus were unreliable for our data. Our background correction method differs from the standard background-subtraction method primarily by the multiplication of each sample by a scaling factor prior to background subtraction. This does have the effect of dramatically shifting the intensity values of some samples (when the range of background intensities among the 27 background probes was low). However, since our method of between-sample normalization also involves a simple scalar multiplication (described below), this effect is removed in later processing steps, and thus our background correction has little effect on values much above background.

Step 4: Normalize samples

We identified three potential control genes (*CyclintT, RBM8A,* and *Cog1*) on the basis of the constancy in expression level across multiple developmental stages in a previous microarray study [25]. After obtaining our DASL results, we checked the viability of each of these three potential control genes following the method of Vandesompele and colleagues [26]. Their method ranks the validity of a gene as a potential control gene with respect to the standard deviation in expression ratio to all other genes in the study. On the basis of this criterion, *Cog1* failed as a control gene, and *CyclinT* appeared marginal. We therefore conducted qPCR studies (described below) using absolute embryo number for normalization. On the bases of these results (data not shown) we eliminated *CyclinT* as a control gene and normalized instead to four

probes that measured the expression of *RBM8A*, which behaved very well in our control qPCR analyses. On the basis of comparisons between our data and those of other studies (below), we feel very confident about *RBM8A* as a normalizer. Quantitative RT-PCR (qPCR) measurements were conducted on an ABI PRISM 7000 (Applied Biosystems). Each reaction consisted of: 15 µl 2X ABGene Absolute qPCR SYBR® Green Mix, 0.75 µl for each primer (10 µM), 1 µl of cDNA template, and PCR quality water to reach a total volume of 30 µl. The PCR program used for all reactions was: 95˚C for 5 min, 40 cycles of 95˚C for 15 sec and 54˚C for 30 sec, followed by a melt curve from 60 to 95˚C. Ct values were determined using the CalQPlex setting. For each primer pair, a standard curve was set up over a twelve point, factor-of-two dilution series to determine the efficiency and working Ct range. We ran each sample for each gene in triplicate wells. Control samples were run in technical duplicates. Only measurements with standard deviations less than 0.4 Ct across replicates were used. To convert the raw Ct expression into normalized relative expression, we utilized a modified delta-delta Ct method [26].

Step 5a: Remove probes and designate gene clusters

We hand-curated the set of 3-6 probes for each gene by assessing the dynamic range and developmental time course for each over the 8 stages measured, specifically examining the strength and linearity of the correlation between each pair of probes. Five genes were removed for which no two probes were correlated over time, and all probes with expression levels that seldom rose above background. For 9 other genes, the set of probes consistently detected above background could be divided into 2-3 groups that were poorly correlated with each other. In most cases, these groups distinguished different exons and thus likely measured alternatively spliced transcripts, although errors in gene annotation cannot be ruled out. In total, 144/384 probes were removed for these reasons. Sets of highly correlated probes for a single gene were designated as “gene clusters”. Several genes had multiple clusters. Based on the observation that these different clusters corresponded to probes marking different exons, we hypothesize that these clusters represent different splice variations. This hypothesis is supported by the observation that the clusters for *otx* correspond to documented splice forms [27]. For analyses

of correlations and covariances between genes, only the first clusters (those containing the most highly correlated probes) were used. Secondary clusters were used only to evaluate changes in parental effects over development. Our nomenclature reflects cluster assignments (*e.g.* otx_2 represents the second cluster of otx, not to be confused with otx_time2 which would represent otx_1 at time point 2). To the extent possible, we demanded that all beads within a single cluster showed a correlation of at least 0.8 over the course of development.

Step 5b: Remove probes due to polymorphisms

Because sea urchins populations contain high levels of genetic diversity [28], we expected some probes to fail in some individuals due to polymorphisms in their region of hybridization. We detected hybridization problems by measuring the "expression" of each probe in a gDNA sample collected from each of the 36 replicated cultures. For each probe in each sample, we calculated the MAD from the overall probe median. 30 Probes with especially large variances (> 2 * MAD of all other probe's MADs) were considered too noisy to evaluate and were removed (Figure S9). Hybridization problems due to polymorphisms should affect samples with the same genetic background similarly. In some cases, we did observe significant correlations between gDNA "expression" and RNA expression, which are likely to be caused by common hybridization differences among samples with different genomic sequences. Thus, for each probe, we collected the MAD values for all pairs of replicates of each of the cultures. Probes in any culture in which both replicate gDNA measurements were more than 2 MADs from the probe median in the same direction (both below the median, or both above) were removed (Figure S9). On average, ~2 probes (max = 13) were removed from each sample. This strategy removed nearly all correlations between the "expression" level of gDNA and gene expression levels.

Step 5c: Remove batch effects

The DASL assay on BeadStation is run in 96-well plates. Each plate is processed separately, and may use a different lot of reagents. Thus, consistent differences between measured expression values of comparable samples in different batches are likely to be technical in origin. We assigned the samples in each 96-well plate to optimize the detection of batch effects: the

two replicate cultures at each developmental stage were split into two different batches, and run together with all other samples collected from the same developmental stage. Thus, to account for batch effects, we collected all samples from each stage, and for each individual probe calculated the mean measured expression of that probe in each batch. We then added (or subtracted) a value to each sample on each plate to make all the batch-means equal.

Step 5d: Summarize individual probe values

We averaged the remaining probe measures into a single value for each gene. For the 9 genes in which we assigned probes to separate clusters, we averaged each group of probes separately, and treated the two clusters as different genes in downstream analyses (see above). When we removed a particular probe from a sample due to irregularities in the gDNA measurements, we did not average the remaining probes, but treated the gene as missing data. Different probes targeting the same transcript often reported different average intensities, even when the intensities were highly correlated. Thus averaging different subsets of probes would lead to biased transcript expression estimates. Here, as elsewhere, we were particularly concerned not to induce artifactual biases that might be correlated with particular families, as these biases would affect our downstream genetic analyses. As hybridization problems due to sequence polymorphisms under particular probes would likely affect all cultures from the same parents, we chose to remove the whole transcript from analysis whenever any of its probes were in question. For most analyses, the expression values for individual genes were first scaled by the mean expression of that gene at that stage. This was primarily done so that the square roots of the variance estimates resulting from our quantitative genetics models (see below) could be read in the same scale as the mean expression.

Expression validation

Following pre-processing steps, we compared the resulting gene expression values to qPCR data from our own lab in addition to gene expression measurements obtained by other labs using a variety of platforms. For a random subset of the samples at stages 2 and 6, we

measured the expression levels of four genes (clusters B-catenin_1, Otx_1, SM50_1, and Endo16_1). We compared the ratio of the expression of each of these genes to mean of the others from our qPCR data and our un-normalized DASL data (Figure S10). The correlation between datasets has a Spearman Rho of 0.62 ($p = 9.88 \times 10^{-10}$), comparable to those reported elsewhere for comparisons between platforms [29,30]. As a second check, we compared our normalized expression values to normalized whole-genome NimbelGen array data [25]. As is standard for microarray data, the Wei et al. data have been quantile normalized so that comparisons between their data and ours tests both our ability to detect changes in gene expression and our normalization scheme. For three time points in which the data were comparable (eggs, time 2 and time 6) we compared the expression of all of our genes that were measured on the array (Figure S11). The overall correlation is quite high (Spearman's Rho = .759, $p < 2.2 \times 10^{-16}$). Our plot of the comparison between the two datasets as well as histograms of the distribution of expression values on the two platforms (Figure S12) reveal an important merit of the DASL platform: sensitivities across the range of expression values found for early developmental genes are better measured on the DASL platform than by traditional microarrays. Our data provide a comprehensive view of how the expression of individual genes changes over development. For 15 of these genes, a similar time series was also obtained using qPCR and a different normalizing gene [31]. The two datasets show substantial qualitative agreement for 12 of the 15 genes (Figures S11 and S12) (note that the datasets come from embryos raised at different temperatures: ours at 12°C and those of Howard-Ashby et al. at 15°C). In addition, we compared our results to another dataset generated using the Nanostring platform [32], where 42 genes overlapped with our dataset. In seven cases the probes used for nanostring measurements targeted UTRs, which can be highly genetically polymorphic in *S. purpuratus*, or targeted an ambiguous gene annotation (for example, our probe for the *Lim1* targets the annotation labeled "Lim1" where as the Nanostring probe targets the paralogous annotation labeled "Lmx1"). The overall correlation between the datasets is reasonable (Spearman Rho =

0.71; p-value < 2.2×10^{-16}), but highly variable among genes. For some genes, the correlation between the datasets is quite high. For example, for the genes *Hex* and *Nk.1* the Spearman Rho = 0.9933. For other genes, however, the correlations are lower, and in one case (*HesC*) slightly negative. This discrepancy stems from a number of sources. For example, *HesC* shows little variability in either dataset. In most cases, however, discrepancies appear to be the result of a phenomenon that we noticed among our own probes for the same gene, namely that different probes targeting the same transcript can show different results. This highlights the importance of using multiple probes for interrogating gene expression levels. Finally, we note a high consistency among technical replicates on the DASL platform. Among three pairs of technical replicates, no correlation was less than 95%.

SUPPLEMENTAL REFERENCES

1. Strathmann MF (1987) Reproduction and development of marine invertebrates of the northern Pacific coast: data and methods for the study of eggs, embryos, and larvae. Seattle, WA: University of Washington Press.
2. Hart MW, Scheibling RE (1988) Comparing shapes of echinoplutei using principal components analysis, with an application to larvae of Strongylocentritus droebachiensis. In: Burke RD, Mladenov PV, Lambert P, Parsley RL, editors. Echinoderm Biology. Rotterdam: A.A. Balkema. pp. 277-287.
3. Bertram DF, Strathmann RR (1998) Effects of maternal and larval nutrition on growth and form of planktotrophic larvae. Ecology 79: 315-327.
4. Poorbagher H, Lamare MD, Barker MF (2010) The relative importance of parental nutrition and population versus larval diet on development and phenotypic plasticity of *Sclerasterias larvae*. Journa of the Marine Biological Association of the United Kingdom 90: 527-536.
5. Klingenberg C, Zaklan S (2000) Morphological integration between developmental compartments in the Drosophila wing. Evolution 54: 1273-1285.
6. Rohlf FJ, Corti M (2000) Use of two-block partial least-squares to study covariation in shape. Syst Biol 49: 740-753.
7. Lynch M, Walsh B (1998) Genetics and analysis of quantitative traits. Sunderland, Mass.: Sinauer. xvi, 980 p. p.
8. Comstock RE, Robinson HF (1948) The components of genetic variance in populations of biparental progenies and their use in estimating the average degree of dominance. Biometrics 4: 254-266.
9. Hadfield JD (2010) MCMC Methods for Multi-Response Generalized Linear Mixed Models: The MCMCglmm R Package. Journal of Statistical Software 33: 1-22.
10. Gelman A (2006) Prior distributions for variance parameters in hierarchical models. Bayesian Analysis 1: 515-533.
11. Ayroles JF, Carbone MA, Stone EA, Jordan KW, Lyman RF, et al. (2009) Systems genetics of complex traits in Drosophila melanogaster. Nature Genetics 41: 299-307.
12. Vazquez AI, Bates DM, Rosa GJ, Gianola D, Weigel KA (2010) Technical note: an R package for fitting generalized linear mixed models in animal breeding. Journal of Animal Science 88: 497-504.
13. Gilmour AR, Gogel BJ, Cullis BR, Thompson R (2009) ASReml User Guide Release 3.0. Hemel Hempstead, UK: VSN International Ltd.
14. Wong WC, Loh M, Eisenhaber F (2008) On the necessity of different statistical treatment for Illumina BeadChip and Affymetrix GeneChip data and its significance for biological interpretation. Biology Direct 3: 23.
15. Gentleman RC, Carey VJ, Bates DM, Bolstad B, Dettling M, et al. (2004) Bioconductor: open software development for computational biology and bioinformatics. Genome Biology 5: R80.
16. Dunning MJ, Smith ML, Ritchie ME, Tavare S (2007) beadarray: R classes and methods for Illumina bead-based data. Bioinformatics 23: 2183-2184.
17. Cairns JM, Dunning MJ, Ritchie ME, Russell R, Lynch AG (2008) BASH: a tool for managing BeadArray spatial artefacts. Bioinformatics 24: 2921-2922.
18. Dombkowski AA, Thibodeau BJ, Starcevic SL, Novak RF (2004) Gene-specific dye bias in microarray reference designs. FEBS Lett 560: 120-124.
19. Yang YH, Dudoit S, Luu P, Lin DM, Peng V, et al. (2002) Normalization for cDNA microarray data: a robust composite method addressing single and multiple slide systematic variation. Nucleic Acids Research 30: e15.
20. Irizarry R, Hobbs B, Collin F, Beazer-Barclay YD, Antonellis KJ, et al. (2003) Exploration, normalization, and summaries of high density oligonuleotide array probe level data. Biostatistics 4: 249-264.

21. Du P, Kibbe WA, Lin SM (2008) lumi: a pipeline for processing Illumina microarray. Bioinformatics 24: 1547-1548.
22. Dunning MJ, Barbosa-Morais NL, Lynch AG, Tavare S, Ritchie ME (2008) Statistical issues in the analysis of Illumina data. BMC Bioinformatics 9: 85.
23. Ding LH, Xie Y, Park S, Xiao G, Story MD (2008) Enhanced identification and biological validation of differential gene expression via Illumina whole-genome expression arrays through the use of the model-based background correction methodology. Nucleic Acids Res 36: e58.
24. Lin SM, Du P, Huber W, Kibbe WA (2008) Model-based variance-stabilizing transformation for Illumina microarray data. Nucleic Acids Research 36: e11.
25. Wei Z, Angerer RC, Angerer LM (2006) A database of mRNA expression patterns for the sea urchin embryo. Developmental Biology 300: 476-484.
26. Vandesompele J, De Preter K, Pattyn F, Poppe B, Van Roy N, et al. (2002) Accurate normalization of real-time quantitative RT-PCR data by geometric averaging of multiple internal control genes. Genome Biology 3: RESEARCH0034.
27. Li X, Chuang CK, Mao CA, Angerer LM, Klein WH (1997) Two Otx proteins generated from multiple transcripts of a single gene in Strongylocentrotus purpuratus. Developmental Biology 187: 253-266.
28. Sodergren E, Weinstock GM, Davidson EH, Cameron RA, Gibbs RA, et al. (2006) The genome of the sea urchin Strongylocentrotus purpuratus. Science 314: 941-952.
29. Wang Z, Gerstein M, Snyder M (2009) RNA-Seq: a revolutionary tool for transcriptomics. Nature Reviews Genetics 10: 57-63.
30. Babbitt CC, Fedrigo O, Pfefferle AD, Boyle AP, Horvath JE, et al. (2010) Both noncoding and protein-coding RNAs contribute to gene expression evolution in the primate brain. Genome Biology and Evolution 2: 67-79.
31. Howard-Ashby M, Materna SC, Brown CT, Chen L, Cameron RA, et al. (2006) Gene families encoding transcription factors expressed in early development of Strongylocentrotus purpuratus. Developmental Biology 300: 90-107.
32. Materna SC, Nam J, Davidson EH (2010) High accuracy, high-resolution prevalence measurement for the majority of locally expressed regulatory genes in early sea urchin development. Gene Expression Patterns 10: 177-184.
